# Postprandial glycemic and lipidemic effects of black rice anthocyanin extract fortification in foods of varying macronutrient compositions and matrices

**DOI:** 10.1038/s41538-023-00233-y

**Published:** 2023-11-01

**Authors:** Sean Jun Leong Ou, Dimeng Yang, Hanny Putri Pranata, E Shyong Tai, Mei Hui Liu

**Affiliations:** 1 Division of Endocrinology, Department of Medicine, Yong Loo Lin School of Medicine, National University of Singapore, 14 Medical Drive, Singapore, 117599 Singapore; 2 Department of Food Science and Technology, National University of Singapore, 3 Science Drive 3, Singapore, 117543 Singapore; 3 Division of Endocrinology, University Medicine Cluster, National University Hospital, 5 Lower Kent Ridge Road, Singapore, 119074 Singapore

**Keywords:** Endocrinology, Nutrition

## Abstract

Anthocyanin (ACN) fortification of commonly consumed foods is significant as a dietary strategy against the development of metabolic complications by delivering ACNs at high doses. However, its bioactivity and translated metabolic effects in the presence of varying food matrices and macro-constituents is particularly unclear. This end-to-end study investigates the metabolic effects of black rice ACN extract (BRAE) fortification—from in-vitro enzyme inhibitory activities and digestibility, to downstream in vivo impacts on GI, postprandial glycemia and lipidemia. The in vivo effects were investigated in two separate crossover randomised controlled trials (RCT) of 24 healthy participants each—the first RCT determined the postprandial blood glucose, insulin, and ACN bioavailability to a starch-rich single food over 2 h, while the second RCT determined the postprandial blood glucose, insulin, lipid panel, and lipoprotein particles and subfractions to a starch- and fat-rich composite meal over 4 h. In-vitro findings confirmed the inhibitory activities of major black rice ACNs on carbohydrases (*p* = 0.0004), lipases (*p* = 0.0002), and starch digestibility (*p* < 0.0001). in vivo, a 27-point mean GI reduction of wheat bread was observed with BRAE fortification, despite a non-significant attenuation in postprandial glycemia. Conversely, there were no differences in postprandial glycemia when fortified bread was consumed as a composite meal, but acute lipid profiles were altered: (1) improved plasma HDL-c, ([0.0140 mmol/L, 95% CI: (0.00639, 0.0216)], *p* = 0.0028), Apo-A1 ([0.0296 mmol/L, 95% CI: (0.00757, 0.0515)], *p* = 0.0203), and Apo-B ([0.00880 mmol/L, 95% CI: (0.00243, 0.0152)], *p* = 0.0185), (2) modified LDL and HDL subfractions (*p* < 0.05), and (3) remodelled lipid distributions in HDL and LDL particles. This end-to-end study indicates the potential of ACN fortification in GI reduction and modulating postprandial lipoprotein profiles to starch- and fat-rich composite meals.

## Introduction

Energy-dense diets rich in rapidly digestible carbohydrates and fats can lead to the pathological development of uncontrolled hyperglycemia and obesity, which have major roles in the pathophysiology of T2D, CVD, and other metabolic complications. Dietary modifications have been recognized as a strategy against the development and regression of metabolic disorders^1,2^, with one such strategy being increasing the consumption of plant-derived foods rich in anthocyanins (ACN) due to its suggested role in mitigating risks in T2D and CVD, on top of its plethora of associated health benefits^3^. In the pathology of cardiometabolic disorders, increasing dietary ACN intake has beneficial effects on glucose and lipid metabolism^4–6^. In-vitro, ACNs have acute inhibitory effects on α-amylase, α-glucosidase, and lipase^7–9^ which decrease the rates of starch and lipid hydrolysis. On the cellular level, ACNs affect the expression of various genes involved in carbohydrate and lipid metabolism^4,10^. The supplementation of ACNs in our diets has also demonstrated improvements in glucose and lipid profiles in-vivo^4–6^.

Black rice is a widely studied source of ACNs due to its popularity in Asia where it is widely cultivated^11^. Unlike other sources of ACN such as blueberries which are not as widely produced and accessible in Asia^12^, pigmented rice including black rice is native to Asia and frequently consumed as staple foods or for therapeutic purposes^11^. Black rice and its extracts have also been used in some food applications to improve the functionality and nutritional quality of starch-based foods, often with the objective of reducing starch digestibility^8,13–15^. The anti-glycemic and anti-lipidemic effects of ACNs, and the major ACN constituents of black rice: cyanidin-3-glucoside (C3G), cyanidin-3-rutinoside (C3R), and peonidin-3-glucoside (P3G), have been assessed in separate in-vitro and in-vivo animal studies. Collectively, these studies on black rice ACNs have demonstrated improved glycemic and lipidemic profiles^16,17^, as well as positive effects on enzyme inhibitory and key metabolic pathways^17–19^.

To date, ACN-food fortification studies have largely only characterized the use of ACNs in foods in-vitro^8,13,15^, while human intervention studies have largely investigated the effects among individuals with compromised baseline metabolism^20–23^, or from the long-term consumption of naturally °Ccurring ACN-rich foods and supplementation^20–23^. There has been an existing lack of end-to-end assessments on the nutritional properties of ACN fortification from within common composite foods that form our daily diets, to its translated effects within healthy humans, where its findings carry greater translational relevance and may work to increase the repertoire of functional food delivery systems. Additionally, the limited acute human intervention studies on the postprandial effects of ACNs and ACN-fortified foods among healthy individuals calls for greater clarity on the efficacy of ACN co-consumption with different foods. This is significant as food matrices may interact with ACNs to alter the latter’s intended effects^14,24^. The assessment of postprandial metabolic responses is also representative of the non-fasted state experienced in most of our waking hours and are increasingly important predictors of metabolic disorders^25,26^. However, the postprandial effects of ACN fortification in mixed meals have not been examined systematically.

As such, this investigation was designed to evaluate, in two parts, the effects of black rice ACN extract (BRAE) fortification on glycemic index (GI) and the postprandial glycemic responses to a starch-rich single food; followed by the postprandial glycemic and lipidemic responses to a starch and fat-rich composite meal, among healthy individuals.

## Results

### Baseline characteristics

**Fig. 1:**
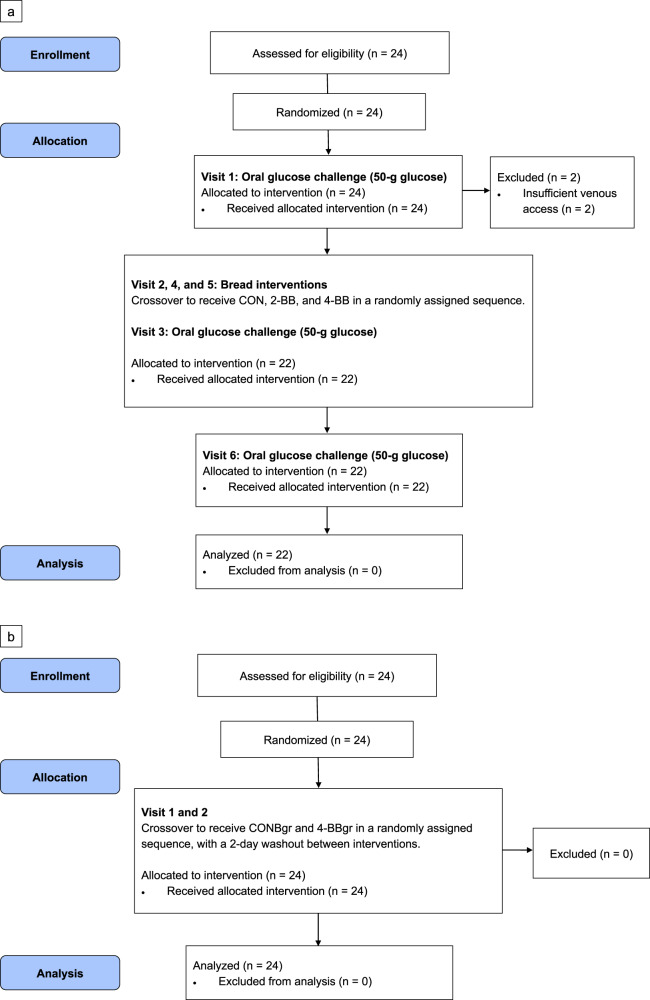
Consolidated Standards of Reporting Trials (CONSORT) flow diagram. Flow diagram of the progress through the phases of randomised controlled trials. **a** *Bread* trial, **b** *Burger* trial.

Twenty-four participants were enrolled and randomized into the intervention arms of each *Bread* and *Burger* trials (Fig. 1). In the *Bread* trial, 22 of 24 participants had completed all six study visits and were analyzed (Fig. 1a). All participants had completed both study visits in the *Burger* trial and were analyzed (Fig. 1b). Two participants had withdrawn from the *Bread* trial due to insufficient venous access during phlebotomy. None of the participants had reported any adverse events from the consumption of the test foods. The baseline characteristics of the participants are summarized in Table 1.

**Table 1:** Baseline characteristics of healthy participants who completed the (A) *Bread*, and (B) *Burger* trials.

Variable	Mean (SD)	Range
(A) *Bread* trial		
Gender, M/F	7/15	-
Age, year	25 (6)	22—52
BMI, kg m^−2^	21.6 (2.5)	18.3—25.8
Systolic BP, mm Hg	112.5 (11.3)	92.1—143.7
Diastolic BP, mm Hg	65.3 (6.7)	54.8—83.0
Fasting glucose, mmol/L	4.42 (0.62)	3.9—5.0
Fasting insulin, mU/L	6.19 (3.12)	1.7—10.9
(B) *Burger* trial		
Gender, M/F	12/12	-
Age, year	29 (11)	21—54
BMI, kg m^−2^	21.3 (1.7)	18.7—25.0
Systolic BP, mm Hg	111.9 (8.9)	89.5—125.3
Diastolic BP, mm Hg	71.1 (7.7)	58.7—83.2
Fasting glucose, mmol/L	4.45 (0.26)	4.1—4.7
Fasting insulin, mU/L	4.61 (2.50)	1.7—10.9
Fasting TG, mmol/L	0.67 (0.31)	0.4—1.9
Fasting TC, mmol/L	4.27 (0.65)	3.9—5.7
Fasting HDL-c, mmol/L	1.69 (0.41)	0.9—2.4
Fasting LDL-c, mmol/L	2.28 (0.47)	1.6—2.7

*BMI* body mass index, *BP* blood pressure, *TG* triglycerides, *TC* total cholesterol, *HDL-c* high-density lipoprotein, *LDL-c* low-density lipoprotein.

## Bread trial

### Inhibition of in-vitro starch hydrolase activity

To confirm the carbohydrase-inhibitory properties of major black rice ACNs, we examined their inhibitory activities on the key starch hydrolases of a simulated digestion model. The inhibitor assay plots of major black rice ACN species are represented in Supplemental Fig. 3. Acarbose, an anti-glycemic drug, was used as a reference due to its effectiveness as a starch hydrolase inhibitor^27^. As shown in Supplemental Fig. 3, we observed a dose–response retardation of starch hydrolase activities by the major black rice ACNs.

In addition, when quantified and expressed in IC_50_ (Table 2), the ACNs had varying inhibitory efficacies against different starch hydrolases, of which C3G had the highest inhibitor activity against α-amylase while C3R best inhibited α-glucosidase.

**Table 2:** IC_50_ of inhibitors on starch hydrolases.

Inhibitor	IC_50_ (μM)	
	α-amylase	α-glucosidase
Acarbose	6.0 (0.3)	2.9 (0.1)
C3G	364 (22)	1116 (3)
P3G	649 (5)	1343 (40)
C3R	483 (26)	904 (3)

*C3G* cyanidin-3-glucoside, *P3G* peonidin-3-glucoside, *C3R* cyanidin-3-rutinoside. Values are represented as means and standard deviations in parentheses.

### Inhibition of in-vitro starch digestibility in a black rice anthocyanin extract-fortified, single high-carbohydrate food

To confirm the carbohydrase-inhibiting properties of BRAE in HC food matrices, the starch digestion profiles of test foods were evaluated using simulated gastrointestinal digestion models (Supplemental Fig. 4). An inverse dose-response was observed between bread digestibility and BRAE fortification. Fortification at 4% concentration produced a significant IAUC reduction of 12.3% in 4-BB relative to CON (Table 3). Similarly, the kinetic rate constant (*k*) obtained from the fitted starch hydrolysis curve reflected a 12.6% reduction for 4-BB (Table 3).

**Table 3:** Effects of BRAE enrichment on starch digestibility in an HC food matrix.

Parameter	CON	2-BB	4-BB	*p*
C_mean_ (g/100 g sample)	2.62 (0.5)	2.44 (0.4)	2.29 (0.4)	0.866
IAUC (g/100 g sample × min)	810 (5)^a^	749 (2)^b^	710 (2)^c^	**<0.0001**
*k*	0.00830 (0.0002)^a^	0.00712 (0.00002)^b^	0.00662 (0.0003)^c^	**0.004**

*p* HC-test meal *p* value for the observed differences between CON, 2-BB, and 4-BB groups analysed with a one-way ANOVA and Tukey’s multiple comparisons test, *C*_mean_ mean glucose concentration; *IAUC* incremental area under curve, *k* kinetic constant determined by fitting the digestibility data to the first-order equation, ${C}_{t}={C}_{0}+({C}_{\infty }-{C}_{0})(1-{e}^{-{kt}})$. Values are represented as means and standard error of means in parentheses. Superscript letters indicate statistical differences within rows. Statistical significance was determined at *p* < 0.05 and is represented in bold.

Overall, our in-vitro analyses demonstrate that BRAE fortification of HC single foods, such as in wheat bread, suppressed starch hydrolysis due to the carbohydrase-inhibiting properties of black rice ACNs. From our findings, we hypothesized a potential in-vivo anti-glycemic effect with the consumption of BRAE-fortified HC foods.

### Postprandial glycemic responses to a black rice anthocyanin extract-fortified, single high-carbohydrate food

To assess the in-vivo glycemic-lowering potential of BRAE delivered through HC single foods, we investigated the postprandial glycemic and insulinemic responses to BB over the 2-h trial duration, as presented in Table 4 and Supplemental Fig. 5.

**Table 4:** Changes in measures of postprandial glycemia in response to the 50-g glucose challenge, and 50-g ACHO equivalent HC test meals in the *Bread* (*n* = 22) trial.

*Bread* Trial				
	CON	2-BB	4-BB	*p*
Mean glucose (mmol/L)	1.13 (0.77)^ab^	1.26 (0.79)^a^	1.04 (0.66)^b^	**0.050**
Mean IAUC_0–120_ (mmol/L*min)	159.05 (64)	165.7 (82)	145.1 (96)	0.416
Mean *P*_max_ (mmol/L)	2.42 (0.7)	2.55 (0.9)	2.13 (0.7)	0.213
Mean *T*_max_ (min)	36.14 (11)^a^	42.27 (13)^ab^	49.09 (22)^b^	**0.023**
GI	95.11 (94)	94.07 (58)	68.19 (43)	0.2
Mean insulin (mU/L)	30.9 (18)^a^	22.8 (13)^b^	26.5 (16)^ab^	**0.0031**
Mean IAUC_0–120_ (mU/L*min)	4043 (2458)	2941 (1678)	3584 (2052)	0.108
Mean *P*_max_ (mU/L)	58.5 (42)	41.5 (27)	48.6 (28)	0.112
Mean *T*_max_ (min)	41.59 (16)	38.86 (14)	46.36 (16)	0.280

*p* intervention *p* value for the observed differences between CON, 2-BB, and 4-BB groups analysed with a repeated measures one-way ANOVA and Tukey’s multiple comparisons test, *P*_max_ change in peak concentration, *T*_max_ time to reach peak concentration. *P*_max_ and *T*_max_ were determined from the average of the participants’ *P*_max_ and *T*_max_. Values are adjusted for baseline and represented as means and standard deviations in parentheses. Superscript letters indicate statistical differences within rows. Statistical significance was determined at *p* < 0.05 and is represented in bold.

Through our primary analyses, intakes of BB attenuate both 2-h postprandial glycemic and insulinemic profiles (Table 4). However, these improvements were not dose-dependent. Compared to CON, only the 4-BB intervention reported a reduction of postprandial incremental glucose concentrations but was not statistically significant (Table 4). Pairwise comparisons among interventions had indicated significantly lower 2 h glucose concentrations after 4-BB relative to 2-BB (mean difference: −0.224 mmol/L, 95% CI: (−0.0118, −0.437), *p* = 0.0406), but non-statistically significant reductions relative to CON (Table 4). BRAE fortification had also modulated the time to reach peak glucose concentrations, in which 4-BB had observed a significant delay in *T*_max_ relative to CON (mean difference: 13.0 mmol/L, 95% CI: (0.443, 25.5), *p* = 0.0416) (Table 4). However, no statistically significant differences were noted for glucose IAUCs between the CON and the fortified bread.

On the other hand, mean GIs of CON, 2-BB, and 4-BB were calculated to be 95.1, 94.1, and 68.2 respectively, reflecting a drop in the average GI classification from high (>70) to medium (55—70). The GL of CON, 2-BB, and 4-BB are 47.6, 47.0, and 34.1 respectively.

While postprandial incremental insulin concentrations over 2 h were attenuated in response to BB (Table 4), there was no dose-response. Relative to CON, pairwise comparisons showed significantly reduced insulin concentrations after 2-BB (mean difference: −8.09 mU/L, 95% CI: (−2.09,−14.1), *p* = 0.0144), but non-significant reductions after 4-BB (Table 4). No statistically significant differences were also noted for insulin IAUCs between CON and the fortified bread.

Through a secondary analysis, two-way ANOVA revealed no statistically significant tim × intervention interactions for postprandial incremental glucose (F(4.53, 95.11) = 1.45, *p* = 0.218) and insulin concentrations (F(3.46, 72.68) = 1.28, *p* = 0.288) (Supplemental Table 5). Simple main effect analysis showed an expected significant effect of time after ingestion on postprandial incremental glucose and insulin concentrations (*p* < 0.0001 for both), but a non-statistically significant effect of BRAE concentrations on postprandial incremental glucose (*p* = 0.175) and insulin concentrations (*p* = 0.146).

### Anthocyanin bioavailability

With the bioavailability of ACN being one of the key contributors to its eventual bioactivity^21^, we also explored the postprandial bioavailability of the major black rice ACNs (C3G, C3R, P3G), and several of its major phenolic metabolites (PCA, protocatechuic acid; VA, vanillic acid; FA, ferulic acid)^28^, 60 and 120 min after a simple HC meal (Supplemental Fig. 6). While postprandial bioavailability of ACN and its metabolites varied greatly, changes in plasma concentrations peaked at 60 min for all interventions. Metabolites of ACN were found to be in greater concentrations in plasma as compared to their parent ACNs, with PCA accounting for 93.9% of total polyphenols at 60 min peak concentrations. However, changes observed were not dose-dependent. Significant differences were only observed between 60 and 120 min of VA (mean difference: 0.439; 95% CI: 0.0755, 0.803; *p* = 0.0143) and FA (mean difference: 1.08; 95% CI: 0.118, 2.03; *p* = 0.0241) in the 4-BB group.

## Burger trial

### Inhibition of in-vitro starch digestibility and lipase activity in a black rice anthocyanin extract-fortified, high-carbohydrate/high-fat mixed-nutrient meal

Typical diets often comprise multiple food items of varying macronutrients and matrices. To investigate the effects of BRAE fortification on starch digestibility in a common meal setting, we included protein and fat components in the single HC-test food from the *Bread* trial. The decision to utilise only CON and 4-BB for the resultant HC/HF mixed meals (CONBgr and 4-BBgr) was based on the magnitude of GI reduction as 4-BB exhibited the lowest GI. The composition of the HC/HF mixed meal is represented in Supplemental Table 1.

Before the conduct of the in-vivo study, we validated whether the effects of BRAE fortification on starch digestibility remained consistent in mixed-nutrient meals using simulated gastrointestinal digestion models (Supplemental Fig. 7A). At a 4% fortification, 4-BBGr exhibited a retardation in starch digestibility, as reflected by a 16.1% reduction in *k* (Table 5). Supporting the slower digestion with BRAE fortification is a 17.0% reduction in IAUC, relative to CONBgr (Table 5).

**Table 5:** Effects of BRAE enrichment on starch digestibility and lipase activity parameters.

Parameter	CONBgr	4-BBgr	*p*^*2*^
Starch digestibility			
*C*_mean_ (g/100 g sample)	1.92 (0.33)	1.61 (0.28)	0.480
IAUC (g/100 g sample × min)	582 (3.3)^a^	483 (5.3)^b^	**<0.0001**
*k*	0.00589 (0.00049)^a^	0.00425 (0.00011)^b^	**0.030**
Lipase activity			
IAUC (RFU × min)	12517 (566)^a^	4713 (112)^b^	**0.0002**

*p* HC/HF-test meal *p* value for the observed differences between CONBgr and 4-BBgr groups analysed with an unpaired *t* test, *C*_mean_ mean glucose concentration *IAUC* incremental area under curve, *k* kinetic constant determined by fitting the digestibility data to the first-order equation, ${C}_{t}={C}_{0}+({C}_{\infty }-{C}_{0})(1-{e}^{-{kt}})$. Values are represented as means and standard error of means in parentheses. Superscript letters indicate statistical differences within rows. Statistical significance was determined at *p* < 0.05 and is represented in bold.

Several studies have characterized and reported inhibitory effects of ACNs on pancreatic lipase through in-vitro enzymatic studies^9,19^. To extend our understanding of the lipase-inhibitory potential of BRAE, we also explored the inhibitory effects of BRAE fortification on pancreatic lipase in the HC/HF test food matrix. Pancreatic lipase activity was assessed using the lipase substrate, 4-MUO, which is cleaved by lipase to generate the fluorogenic 4-methylymbelliferyl (4-MU). The 4-MUO hydrolysis profiles over 4 h are represented in Supplemental Fig. 7B, which exhibited an overall attenuated generation of 4-MU in the HC/HF beef burger matrix of 4-BBgr, with a significant 62.3% reduction in the 4-MU 4-h IAUC values (Table 5).

Collectively, our in-vitro analyses show that BRAE fortification in an HC/HF mixed-nutrient beef burger matrix had suppressed starch digestibility and pancreatic lipase activity.

### Postprandial glycemic and lipidemic responses to a black rice anthocyanin extract-fortified, high-carbohydrate/high-fat mixed-nutrient meal

Next, we investigated the effects of BRAE fortification on postprandial glycemic and lipidemic responses to the HC/HF test meal comprising BB and a ground beef patty. Postprandial glucose and insulin profiles to the HC/HF test meals over 4 h are presented in Table 6 and Supplemental Fig. 8A. While BRAE fortification had moderate improvements in postprandial glucose and insulin profiles in response to the HC test meals from the *Bread* trial (Table 4), this effect was not detected after the HC/HF test meals from the *Burger* trial (Table 6), despite an equivalent ACHO content.

**Table 6:** Changes in measures of postprandial glycemia in response to the 50-g ACHO equivalent HC/HF test meals in the *Burger* (*n* = 24) trial.

*Burger* Trial			
	CONBgr	4-BBgr	*p*
Mean glucose (mmol/L)	0.494 (0.48)	0.436 (0.51)	0.116
Mean IAUC_0–120_ (mmol/L*min)	83.8 (54)	80.2 (61)	0.762
Mean IAUC_0–240_ (mmol/L*min)	109 (67)	106 (18)	0.86
Mean *P*_max_ (mmol/L)	1.47 (0.61)	1.44 (0.69)	0.8
Mean *T*_max_ (min)	42.5 (11)	45.3 (31)	0.636
Mean insulin (mU/L)	18.5 (12)	17.7 (9.9)	0.571
Mean IAUC_0–120_ (mU/L*min)	2501 (1504)	2320 (1374)	0.621
Mean IAUC_0–240_ (mU/L*min)	4660 (2918)	4649 (2283)	0.986
Mean *P*_max_ (mU/L)	43.4 (32)	41.0 (26)	0.712
Mean *T*_max_ (min)	55.6 (20)^a^	77.5 (51)^b^	**0.0386**

*p* intervention *p* value for the observed differences between CONBgr and 4-BBgr groups analysed with a paired *t* test, *P*_max_ change in peak concentration, *T*_max_ time to reach peak concentration. *P*_max_ and *T*_max_ were determined from the average of the participants’ *P*_max_ and *T*_max_. Values are adjusted for baseline and represented as means and standard deviations in parentheses. Superscript letters indicate statistical differences within rows. Statistical significance was determined at *p* < 0.05 and is represented in bold.

In contrast, we observed differential 4 h-postprandial lipid profiles in response to HC/HF test meals in the *Burger* trial (Table 7 and Supplemental Fig. 8B). Differences in postprandial incremental triglyceride, total cholesterol, and LDL-c concentrations between interventions were non-significant (Table 7). However, changes in HDL-c and apolipoprotein (Apo) concentrations were significantly different between interventions (HDL-c mean difference = 0.0140 mmol/L, 95% CI: (0.00639, 0.0216), *p* = 0.0028; Apo-A1 mean difference = 0.0296 mmol/L, 95% CI: (0.00757, 0.0515), *p* = 0.0203; Apo-B mean difference = 0.00880 mmol/L, 95% CI: (0.00243, 0.0152), *p* = 0.0185).

**Table 7:** Changes in measures of postprandial lipidemia in response to 50-g ACHO equivalent HC/HF test meals in the *Burger* trial (*n* = 24).

		CONBgr	4-BBgr	*p*
TG	Mean net AUC_0–240_ (mmol/L*min)	83.7 (64)	82.1 (65)	0.83
	Mean TG_0–240_ (mmol/L)	0.231 (0.23)	0.224 (0.23)	0.252
TC	Mean net AUC_0–240_ (mmol/L*min)	−12.7 (34)	−10.0 (21)	0.665
	Mean TC_0–240_ (mmol/L)	−0.046 (0.039)	−0.039 (0.033)	0.299
HDL-c	Mean net AUC_0–240_ (mmol/L*min)	−11.2 (18)	−7.96 (15)	0.511
	Mean HDL_0–240_ (mmol/L)	−0.036 (0.023)	−0.022 (0.025)	**0.0028**
LDL-c	Mean net AUC_0–240_ (mmol/L*min)	−39.9 (41)	−40.9 (35)	0.89
	Mean LDL_0–240_ (mmol/L)	−0.116 (0.098)	−0.125 (0.094)	0.33
Apo-A1	Mean net AUC_0–240_ (mmol/L*min)	−12.7 (16)	−4.92 (15)	0.0837
	Mean Apo-A1_0–240_ (mmol/L)	−0.045 (0.031)	−0.015 (0.022)	**0.0203**
Apo-B	Mean net AUC_0–240_ (mmol/L*min)	−3.14 (6.0)	−0.813 (6.5)	0.201
	Mean Apo-B_0–240_ (mmol/L)	−0.011 (0.008)	−0.003 (0.004)	**0.0185**
Apo-B:Apo-A1	Mean ratio	0.458 (0.0053)	0.467 (0.0045)	**0.0008**

*p* intervention *p* value for the observed differences between CONBgr and 4-BBgr groups analysed with a paired *t* test. *TG* triglycerides, *TC* total cholesterol, *HDL-c* high-density lipoprotein cholesterol, *LDL-c* low-density lipoprotein cholesterol, *Apo-A1* apolipoprotein-A1, *Apo-B* apolipoprotein-B. Values are adjusted for baseline and represented as means and standard deviations in parentheses. Statistical significance was determined at *p* < 0.05 and is represented in bold.

In a secondary analysis, two-way ANOVA revealed no statistically significant time × intervention interactions for postprandial incremental concentrations of glucose (F(8,184) = 0.80, *p* = 0.599), insulin (F(8, 184) = 1.08, *p* = 0.378), and lipids (TG: F(8, 176) = 0.266, *p* = 0.976; TC: F(8, 176) = 0.481, *p* = 0.869; HDL-c: F(5.81, 128) = 0.407, *p* = 0.869; LDL-c: F(5.08, 112) = 0.740, *p* = 0.597; Apo-A1: F(3.35, 77.03) = 0.767, *p* = 0.529; Apo-B: F(3, 69.19) = 0.421, *p* = 0.739) (Supplemental Table 8). As expected, simple main effects analyses indicated that postprandial incremental concentrations of glucose, insulin, and lipids had varied significantly with time after ingestion (*p* < 0.0001 for all). However, simple main effects analyses also indicated that BRAE fortification lacked a statistically significant effect on postprandial incremental concentrations of glucose (*p* = 0.466), insulin (*p* = 0.735), and lipids (TG: *p* = 0.776; TC: *p* = 0.741; HDL-c: *p* = 0.425; LDL-c: *p* = 0.759; Apo-A1: *p* = 0.082; Apo-B: *p* = 0.204).

### Postprandial changes in lipoprotein particles and subclasses to a black rice anthocyanin extract-fortified, high-carbohydrate/high-fat mixed-nutrient meal

Noting the significant differences in HDL-c and apolipoproteins (Table 7), we further explored the modulatory effects of black rice ACNs on lipid metabolism by assessing acute changes in profiles of lipoprotein particles and their subclasses (Table 8 and Supplemental Fig. 9). Between interventions, changes in 4-h mean particle concentrations were significantly different (VLDL mean difference = 5.99 × 10^−7 ^mmol/L, 95% CI: (3.43 × 10^−8^, 1.16 × 10^−6^), *p* = 0.0421; LDL mean difference = 1.73 × 10^−5 ^mmol/L, 95% CI: (4.42 × 10^−6^, 3.01 × 10^−5^), *p* = 0.0203; HDL mean difference = 3.9 × 10^−4 ^mmol/L, 95% CI: (9.96 × 10^−5^, 6.81 × 10^−4^), *p* = 0.0203) (Table 8), while the changes in particle diameters between interventions did not differ (Supplemental Table 9A). Significant changes were also noted among mean subfractional particle concentrations between interventions for XS-VLDL, L-LDL, M-LDL, S-LDL, XL-HDL, L-HDL, M-HDL, and S-HDL (Table 8) from the intake of 4-BBgr. While an attenuation was observed among the postprandial responses in L-LDL, S-LDL, XL-HDL, L-HDL, M-HDL, and S-HDL of the 4-BBgr group; the opposite was detected for XS-VLDL and M-LDL (Table 8). In addition, we observed postprandial differences in lipid composition among LDL and HDL subfractions (Supplemental Table 9B). Within these subclasses, BRAE fortification did not induce differences in postprandial incremental TG concentrations in an HC/HF matrix, but attenuated those of total lipids, PL, TC, CE, and FC (Supplemental Table 9B).

**Table 8:** Changes in particle concentrations of postprandial lipoproteins and their subclasses over 4 h.

Lipoprotein subclasses						
	Mean concentration (mmol/L)			Net AUC (mmol/L*min)		
	CONBgr	4-BBgr	*p*	CONBgr	4-BBgr	*p*
VLDL and subfractions						
VLDL	7.6 × 10^−6^ (5.6 × 10^−6^)	8.19 × 10^−6^ (5.7 × 10^−6^)	**0.0388**	3.13 × 10^−5^ (1.8 × 10^−5^)	3.41 × 10^−5^ (2.6 × 10^−5^)	0.61
CM and XXL-VLDL	2.78 × 10^−7^ (1.9 × 10^−7^)	2.60 × 10^−6^ (2.1 × 10^−7^)	0.489	6.93 × 10^−5^ (3.4 × 10^−5^)	6.40 × 10^−5^ (3.8 × 10^−5^)	0.538
XL-VLDL	5.77 × 10^−7^ (3.6 × 10^−7^)	5.52 × 10^−7^ (3.5 × 10^−7^)	0.304	0.000148 (8.7 × 10^−5^)	0.000143 (1.0 × 10^−4^)	0.720
L-VLDL	1.45 × 10^−6^ (8.8 × 10^−7^)	1.39 × 10^−6^ (8.4 × 10^−7^)	0.103	0.000372 (1.9 × 10^−4^)	0.000358 (2.3 × 10^−4^)	0.707
M-VLDL	1.44 × 10^−6^ (9.6 × 10^−7^)	1.64 × 10^−6^ (1.0 × 10^−6^)	0.0613	6.02 × 10^−6^ (5.2 × 10^−6^)	6.94 × 10^−6^ (7.9 × 10^−6^)	0.608
S-VLDL	2.70 × 10^−6^ (2.0 × 10^−6^)	2.93 × 10^−6^ (2.1 × 10^−6^)	0.115	1.11 × 10^−5^ (6.0 × 10^−6^)	1.23 × 10^−5^ (8.3 × 10^−6^)	0.447
XS-VLDL	1.15 × 10^−6^ (1.3 × 10^−6^)	1.43 × 10^−6^ (1.4 × 10^−6^)	**0.0494**	4.32 × 10^−6^ (7.1 × 10^−6^)	5.64 × 10^−6^ (8.7 × 10^−6^)	0.523
IDL, LDL, and subfractions						
IDL	1.11 × 10^−6^ (1.5 × 10^−6^)	3.93 × 10^−6^ (9.9 × 10^−7^)	0.226	4.07 × 10^−6^ (3.2 × 10^−5^)	1.40 × 10^−6^ (5.2 × 10^−5^)	0.823
LDL	−3.10 × 10^−5^ (2 × 10^−5^)	−1.38 × 10^−5^ (9.9 × 10^−6^)	**0.0203**	−0.00824 (0.010)	−0.00371 (0.0093)	0.0997
L-LDL	−2.34 × 10^−5^ (1.4 × 10^−5^)	−1.57 × 10^−5^ (1.1 × 10^−5^)	**0.0179**	−0.00634 (0.007)	−0.00435 (0.006)	0.209
M-LDL	−3.32 × 10^−6^ (3.1 × 10^−6^)	3.18 × 10^−6^ (3.0 × 10^−6^)	**0.0270**	−0.000812 (0.004)	0.000914 (0.003)	0.0660
S-LDL	−4.29 × 10^−6^ (3.4 × 10^−6^)	−1.22 × 10^−6^ (1.3 × 10^−6^)	**0.0462**	−0.00109 (0.002)	−0.000279 (0.002)	0.104
HDL and subfractions						
HDL	−6.43 × 10^−4^ (4 × 10^−4^)	−0.000253 (1.9 × 10^−4^)	**0.0203**	−0.176 (0.21)	−0.0731 (0.18)	0.0621
XL-HDL	2.85 × 10^−6^ (7.4 × 10^−6^)	−1.19 × 10^−6^ (7.6 × 10^−6^)	**0.0279**	0.000444 (0.003)	−0.000653 (0.003)	0.111
L-HDL	−6.10 × 10^−5^ (6.2 × 10^−5^)	−4.64 × 10^−5^ (6.2 × 10^−5^)	**0.0295**	−0.0186 (0.02)	−0.0149 (0.02)	0.593
M-HDL	−0.000176 (1.2 × 10^−4^)	−5.70 × 10^−5^ (8.2 × 10^−5^)	**0.0203**	−0.0499 (0.06)	−0.0186 (0.06)	0.0636
S-HDL	−0.000409 (2.5 × 10^−4^)	−0.000148 (9.8 × 10^−5^)	**0.0209**	−0.108 (0.1)	−0.0390 (0.1)	**0.0354**

*p* intervention *p* value for the observed differences between CONBgr and 4-BBgr groups analysed with a paired *t* test. *XXL-VLDL* extremely large very low-density lipoproteins, *XL-VLDL* very large very low-density lipoproteins, *L-VLDL* large very low-density lipoproteins, *M-VLDL* medium very low-density lipoproteins, *S-VLDL* small very low-density lipoproteins, *XS-VLDL* very small very low-density lipoproteins, *IDL* intermediate-density lipoprotein, *L-LDL* large low-density lipoprotein, *M-LDL* medium low-density lipoprotein, *S-LDL* small low-density lipoprotein, *XL-HDL* very large high-density lipoprotein, *L-HDL* large high-density lipoprotein, *M-HDL* medium high-density lipoprotein, *S-HDL* small high-density lipoprotein. Values are adjusted for baseline and represented as means and standard deviation in parentheses. Statistical significance was determined at *p* < 0.05 and is represented in bold.

From our secondary analyses, two-way ANOVA indicated no statistically significant time × intervention interactions for postprandial incremental concentrations of lipoproteins and their subfractions (Supplemental Table 9C). Simple main effects analyses had indicated statistically significant, albeit expected, changes in lipoprotein particles and their subfractional concentrations with time after the ingestion of test meals. On the other hand, simple main effects analyses also indicated non-statistically significant intervention effects on the postprandial incremental concentrations of lipoprotein particles and their subfractions, except for S-HDL (*p* = 0.0364) (Supplemental Table 9C).

Collectively, we observed changes in lipoprotein particles, subfractions, and lipid compositions with BRAE fortification which indicate a modulatory effect on low- and high-density lipoproteins by black rice ACNs. Our data suggests a role for ACN and its metabolites on lipoprotein metabolism.

## Discussion

This suite of studies was designed to systematically investigate the modulatory effects of ACNs on postprandial glycemic and lipidemic responses to a single food and a mixed-nutrient meal. We took a mechanistic approach to our investigation, in which we assessed the postprandial glycemic profiles of a single HC-food item in the first clinical trial, then the postprandial glycemic and lipidemic responses to a HC/HF mixed-nutrient meal in the second trial. In each trial, we methodically approached our objectives by: (1) confirming the enzyme inhibitory properties of black rice ACNs; (2) assessing the inhibitory effects of BRAE fortification on starch digestibility through simulated digestion models; and finally (3) investigating the postprandial glycemic and lipidemic profiles to BRAE-fortified single foods and composite meals among healthy individuals. The in-vitro investigations not only confirmed the inhibitory action of black rice ACNs on hydrolases, but also evaluated the modulatory effects of ACN on starch digestibility in the presence of food matrix effects. Acute feeding trials were carried out in this study to elucidate the postprandial effects of ACN on metabolic responses (i.e., glycemic and lipidemic), as an individual’s postprandial metabolic response is predictive of cardiometabolic risk^25,26,29^. We also used realistic meal challenges comprising complex food matrices and food combinations to illustrate the differences in postprandial metabolic outcomes from ACN fortification under different matrix effects. Such a design would increase translational and clinical relevance.

While we show that the incorporation of BRAE into wheat bread had slight effects on postprandial glucose and insulin profiles in an acute feeding setting, the use of BRAE at 4% concentration can reduce the average GI of bread by 27 points. This suggests potential benefits for healthy individuals who may substitute carbohydrate-rich foods with ACN-fortified substitutes of lower GI in their habitual diet. High variability in the measures of postprandial glycemic responses were noted in our study and may have contributed to the lack of statistical significance observed. This is congruent to other studies that attributed this to the inherent high inter- and intra-individual variability among healthy individuals in response to identical foods^30,31^.

Results from our *Burger* trial suggest that the natural complexity of food matrices further blunts the effects of ACNs on the same carbohydrate load. Food matrices are physical domains comprising the physical and chemical interactions with food constituents—a result of food microstructures and nutrients. When compared to free ACN, interactions of ACN within the food matrices affects its functionality, accessibility, and resulting bioactivity^21^. Thus, the lack of differences in postprandial response effects may be contributed by the protein and fat constituents in the *Burger* test meal. However, there has been limited clinical evidence that elucidates the effects of proteins- and lipid-rich food matrices on polyphenol accessibility and bioavailability. Moreover, of the in-vitro and in-vivo studies conducted, results have been inconclusive. Proteins were demonstrated to complex reversibly and non-reversibly with ACNs^32,33^, and can impact ACN bioaccessibility and bioavailability^34–37^. However, studies have suggested differences in binding affinities among ACN species and varying protein matrices^32,33^. Similarly, interactions between lipids and ACNs remain unclear, with incongruence between the in-vitro and in-vivo findings on ACN accessibility^34–37^. Interestingly, lipid-rich meal matrices were observed to selectively influence the bioaccessibility of some ACN species based on structural differences^37^.

Apart from TG, TC, and LDL-c, HDL-c and apolipoproteins were positively influenced by BRAE fortification in the *Burger* trial. Our observations on the effects of ACN on HDL-c and apolipoproteins were largely consistent with a recent study^5^, which had reported favorable changes in HDL-c and Apo-A1 after the acute feeding of ACN-rich blueberries with an energy-dense drink. However, we also noted elevated levels of Apo-B in the 4-BBgr group (Table 7). Apo-A1 is a major component of HDL particles and plays a key role in reverse cholesterol transport through the generation of nascent HDL particles^38^. On the other hand, Apo-B is a structural component of CM (Apo-B48), VLDL, and its metabolites IDL and LDL (Apo-B100). While generally considered atherogenic, the synthesis of Apo-B is essential in the postprandial clearance of TG from CM in the intestines, as well as endogenous TG catabolism from VLDL, LDL, and IDL via lipoprotein lipase^39^. A growing body of evidence suggests a protective role of ACNs against lipid abnormalities, via modulatory mechanisms involving cholesterol synthesis and lipoproteins^4,22,40^. The lack of differences in CM and XXL-VLDL suggests minimal effects of ACNs on exogenous lipid transport from the intestines, and that the increments in Apo-B may be attributed to Apo-B100 instead of Apo-B48 (Table 8). While the exact mechanisms are unclear, acute elevated levels of both apolipoproteins in 4-BBgr also support the hypothesis that ACNs may have exerted a positive effect on processes in hepatic lipid clearance and reverse cholesterol transport (Table 7), which concurs with the observed statistically significant enrichment in mean concentrations of endogenous VLDL, LDL, and HDL particles (Table 8). We speculate this to °Ccur via possible pathways such as the apolipoprotein-mediated assembly of nascent VLDL (Apo-B100) and HDL (Apo-A1).

Existing evidence on postprandial metabolism is limited, not detailed, and will benefit from further studies. From our analysis of lipoprotein particles and their subclasses, we had generally expected lipoprotein particle and subfractional concentrations to change postprandially after the HF intervention as reported in similar studies^41,42^. We also reported ACN-induced differences in postprandial responses of LDL and HDL subfractions, but very limited to no effects on CM and VLDL subfraction profiles (Table 8). Postprandial lipemia is mainly regulated by the production of CM, with the pathophysiology of dyslipidemia attributed to the blood accumulation of TG-rich lipoproteins synthesized by the intestines (CM) and liver (VLDL)^43^. A 2-week administration of polyphenols to obese males had indicated significantly reduced Apo-B48 production^44^, which is indicative of reduced CM synthesis and intestinal lipid clearance. On the other hand, an 8-week diet high in polyphenols noted no changes in Apo-B48, but significantly reduced TG and TC in VLDL^45^. The hypothesis that ACNs may positively influence lipid clearance by elevating hepatic LDL particle levels is supported by the Apo-B (hypothesized to be attributed to Apo-B100) and LDL particle enrichment after the 4-BBgr intervention (Table 7 and Table 8). While we had noted minimal remodeling among VLDL subfractions (M-VLDL and S-VLDL only) which we attribute to the acute nature of our feeding trial, we observed no changes in CM production, which concurs with the absence of change in Apo-B48 responses in the study by Della Pepa, et al.^45^. On the other hand, we show significant modifications to LDL and HDL subfractions which are characterized by increased TG and reduced TC:TG ratios in LDL, as well as increased cholesterol in HDL subfractions. It has been suggested that polyphenols can modulate LDL and HDL TG by inhibiting the cholesterol ester transfer protein (CETP)^6,45^. The increased CETP activity in a postprandial state induces the exchange of cholesterol in HDL, for TG in LDL^45,46^. An inhibition of CETP by ACNs could impair this cholesterol-TG exchange, leading to the greater enrichment of cholesterol in HDL and TG in LDL which was observed in our ACN group (Supplemental Table 9B). The inhibition of CETP-mediated TG enrichment thus inhibits the remodeling of lipid compositions in lipoprotein particles, leading to the lack of change in lipoprotein particle sizes as evident in Supplemental Table 9A. Polyphenols can also improve acute cholesterol efflux and the expression of ATP binding cassette transporter G1 (ABCG1), which mediates the intercellular redistribution of cholesterol; and scavenger receptor-B1 (SR-B1), which facilitates the uptake of cholesterol into HDL^45,47,48^. The greater postprandial responses among HDL cholesterol and subfraction particle concentrations in the ACN group may thus suggest improvements in the reverse cholesterol transport mechanism.

Apart from the enzyme inhibitory functions of ACNs on carbohydrases and lipases, the bioavailability of ACNs and their metabolites are also significant contributors to its downstream bioactivity post-absorption. Although not reported in this acute study, the involvement of purified ACNs in glucose and lipid metabolism such as by suppressing the expression of glucose transporters, improving muscular and hepatic glucose uptake, as well as increasing fatty acid oxidation while reducing lipolysis has been reported in previous in-vitro and mice studies^2,49^—and these may only take effect in-vivo if ACNs or its metabolites are bioavailable in blood. In the *Bread* trial, specific ACNs and phenolic acids were chosen for the current analysis as C3G, C3R, and P3G have been identified as the major ACNs of black rice^50^. Among the phenolic acids, PCA has been identified as a primary metabolite of ACN, while VA and FA have been identified as PCA-derived metabolites^28^. Maximal polyphenolic concentrations in this study were consistent with previous reports of peak plasma concentrations at 0.5–2 h for ACNs, 1–2 h for PCA, and 0.5–1 h for FA and VA^28,51^. We also observed that phenolic acid metabolites were more bioavailable than ACNs in human plasma, which may be due to ACNs undergoing rapid metabolism into its metabolites upon absorption into systemic circulation which contributed to differences in bioavailability^28,51^. Altogether, findings from our study suggest that the health benefits associated with ACNs are mediated first through the direct inhibition of digestive enzymes by ACNs, followed by the conferment of potential effects from its metabolites upon absorption and metabolism.

The strength of this work lies in our mechanistic approach to investigating the efficacy of black rice ACN fortification in the management of postprandial hyperglycemia and hyperlipidemia. Along with the use of realistic meals, this work also boasts greater translational relevance from the measurement of acute postprandial responses. The accumulation of daily metabolic stressors significantly disrupts metabolic homeostasis, and incrementally contributes to disease risks^26^. The postprandial effects of ACNs on lipoprotein subfractions and their lipid composition are relatively unexplored fields of research. Therefore, our ancillary lipoprotein analysis provides insights into the influence of ACNs on these postprandial metabolic effects among healthy individuals. Nevertheless, findings from this analysis should be confirmed through additional clinical studies of similar designs. Mechanistic studies investigating the effects of ACNs and their metabolites on postprandial lipoprotein metabolism can clarify the pathways involved in the observed metabolic changes. Our study was limited as apart from advising participants to avoid ACN-rich or fat-rich diets before the study and during the washout phase, there were no other dietary restrictions applied while the trial was ongoing. It was possible that this lack of dietary control, together with inherent inter-individual variabilities, may have contributed to the large variations in phenolic concentrations during the study visits. Nonetheless, the strength of the current analysis stems from the realistic quantity of ACNs that participants had consumed in the BB interventions (2-BB: 60.3 mg/serving; 4-BB: 127.3 mg/serving). A 100 g serving of blackberries, which was achievable through daily diets, contains about 89 to 211 mg of total ACNs^3^. As such, we considered the ACN content in the BB interventions to be representative of the amount that may be consumed in the average diet.

In conclusion, this study systematically evaluated the anti-glycemic effects of BRAE in both a single HC-food, and an HC/HF mixed-nutrient meal. Our source of ACN was well characterized to confirm the carbohydrase- and lipase-inhibitory properties of major black rice ACNs in-vitro. While changes in postprandial glycemia and lipid panel responses were largely statistically non-significant due to the large variability observed among healthy individuals, we demonstrated a 27-point GI reduction of wheat bread with BRAE fortification, thus suggesting its potential viability in glycemic control. In addition, we showed improvements in LDL and HDL subfractional profiles, as well as modulatory effects on lipoprotein lipid distributions with BRAE enrichment. Substantial heterogeneity was also shown in postprandial polyphenolic profiles, with metabolites of ACN having substantially greater postprandial bioavailability than ACNs. Altogether, results from this study support BRAE fortification as a potentially viable dietary strategy for better glycemic and lipidemic control among healthy individuals. However, the modulatory effects of ACN on postprandial metabolic responses are food-matrix specific, and greater clarity on the impact of dietary factors on its bioactivity is required to understand the full benefits of ACNs on health and disease prevention.

## Methods

### Study design

This study comprised two randomized controlled crossover trials (RCT) designed to evaluate the efficacy of BRAE fortification in improving metabolic responses to standardized single-food and mixed-nutrient meal challenges. Approval from the National Healthcare Group Domain Specific Review Board, Singapore, had been obtained for each trial before the initiation of study procedures. The trials in this study were registered at clinicaltrials.gov as NCT03989674 and NCT04063137.

The first part of the study, henceforth termed the *Bread* trial (Fig. 1a), was primarily designed to assess the modulatory effects on the GI and postprandial glycemic responses from BRAE fortification of wheat bread, which was selected as the high-carbohydrate (HC) meal challenge used in this trial. This RCT was designed with reference to ISO 26642:2010 for GI determination^52^, where participants had to complete six study visits (three references and three interventions) of 2-h durations with 2-day washout periods between visits to allow for the elimination of ACNs before initiating subsequent interventions. Participants were provided with a 50-g oral glucose challenge for reference visits for the GI determination of the fortified and non-fortified wheat bread according to ISO 26642:2010, and were served with one of the three types of wheat bread for intervention visits (CON: no fortification, 2-BB: 2% BRAE/100 g wheat flour 4-BB: 4% BRAE/100 g wheat flour) according to the random intervention sequence of block size 8 they were allocated to (www.randomization.com). Postprandial blood samples for glucose and insulin measurements were obtained over the study duration (fasting blood, 15, 30, 45, 60, 90, and 120 min after the first bite of the test food). The GI of each test food was determined geometrically according to ISO 26642:2010, in which the incremental area under curves (IAUC) of the test foods were expressed as ratios to the mean IAUC of the three oral glucose references.

The second part of the study henceforth termed the *Burger* trial (Fig. 1B), investigated the changes in postprandial glycemic and lipidemic responses to a standardized HC/HF mixed-nutrient composite meal challenge prepared with BRAE-fortified wheat bread. This RCT comprised 2 intervention visits of 4-h durations, with 2-day washout periods between visits to allow for the elimination of ACNs before initiating subsequent interventions. Participants were randomized to start with either of the two test meals (CONBgr or 4-BBgr) according to the random intervention sequence of block size 12 they were allocated to (www.randomization.com). Postprandial blood samples for glucose, insulin, and lipid panel measurements were obtained over the study duration (fasting blood, 15, 30, 45, 60, 90, 120, 180, and 240 min after the first bite of the test meal).

Prior to both RCTs, participants were instructed to fast for 10 h, avoid fat-rich foods and ACN-rich foods for at least 36 h prior to reporting in the morning of their study visit. Participants were also instructed to avoid strenuous exercise and excessive alcohol intake 24 h before the study visit, and to consume dinner in regular portions the evening before to avoid the second meal effect. On the day of the study visit, participants were instructed to rest in a supinated position for 15 min before blood pressure and body weight measurements. Fasting and postprandial blood samples were obtained through a cannula on the arm not used for blood pressure measurements. Test foods/meals in both RCTs were served with 200 mL of plain drinking water, and finished within 12 min, with physical activities kept to a minimum among participants.

### Participants

In each RCT, 24 men and women were recruited via posters and personal communication around the National University of Singapore campus grounds. The participants for both *Bread* and *Burger* trials fulfilled the following inclusion criteria: Asian Chinese, between 21 and 65 years, non-hypertensive (<140/90 mmHg), body mass index (BMI) between 17 and 28 kg/m^2^, normal fasting blood glucose (<6.1 mmol/L). In the *Burger* trial, participants also had to fulfil the following criteria: normal fasting triglycerides (<1.7 mmol/L) and low-density lipoprotein (LDL) cholesterol (<2.6 mmol/L). Participants who smoked, were pregnant, were on regular medication (western or traditional), or had medical history that may affect the interpretation of results were excluded from the study. Written informed consent had been obtained from each participant prior to their enrolment in each study.

### Test meals

The HC test meals of the *Bread* trial comprised wheat bread samples fortified with varying doses of BRAE—control (CON, no fortification), low BRAE dose (2-BB, 2% BRAE/100 g wheat flour), and high BRAE dose (4-BB, 4% BRAE/100 g wheat flour). Bread samples were prepared according to previously described procedures^8^. Bread is a potential delivery vehicle for ACNs as bakery products are among the most commonly consumed carbohydrate foods category globally^53^. Per 100 g of bread flour (13.1% protein, Prima, Singapore), 4 g of sugar (NTUC Fairprice, Singapore), 3 g of shortening (Red Man, Phoon Huat, Singapore), 1.2 g of salt (NTUC Fairprice, Singapore), 1 g of yeast (*Saccharomyces cerevisiae*, S.I. Lesaffre, France), 59 g of deionized water, and varying quantities of BRAE (Shaanxi Bolin Biotechnology Co Ltd, China) were mixed for 6 min using a KitchenAid mixer (5KPM50, Michigan, USA). The dough was left to rest under ambient conditions for 15 min, then divided and manually molded into 100 ± 1 g boules. The dough boules were proofed for 70 min at 40 °C and 85% relative humidity, before they were baked for 8 min at 200 °C in a convection oven. The wheat bread samples were formulated to provide 50 g available carbohydrates, 2.5 g fat, and 8 g protein, as determined by proximate analysis (Supplemental Table 1). As determined by high-performance liquid chromatography (HPLC), the total ACN content (in C3G equivalents) that had been incorporated was quantified to be 60 mg/100 g bread and 127 mg/100 g bread for 2-BB and 4-BB, respectively (Supplemental Table 1). ACN content at these concentrations of BRAE incorporation was determined to be comparable to that in 100 g blackberries (89–211 mg), which was an achievable quantity through our daily diet^3^. Furthermore, a similar study had previously determined the use of black rice extract of a comparable composition above 4% fortification levels to yield unfavorable quality and textural changes in fortified bread, of which rendered the latter incomparable to unfortified wheat bread^8^.

HC/HF mixed meal challenges in the subsequent *Burger* trial were served in the form of a beef burger, comprising CON or 4-BB (CONBgr, no fortification; 4-BBgr, 4% BRAE/100 g wheat flour) as the source of carbohydrates, 150 g ground beef patty (912253CS, Carne Meats, Indoguna, Singapore) as a source of protein and fat, and 6 g mayonnaise (Kewpie Corp, Tokyo, Japan) as a source of fat. Frozen ground beef patties were left to thaw in a refrigerator the night before and were fully cooked at 200 °C for 20 min in a convection oven in the morning of the study visit. The beef burgers were formulated to provide 50 g available carbohydrates (28% energy), 33 g protein (18% energy), and 43.8 g fat (54% energy), as determined by proximate analysis (Supplemental Table 1).

### Proximate analysis of test meals

Proximate analysis of test meals were performed according to the methods described by the Association of Official Analytical Chemists (AOAC)^54^.

To determine the fat content in test meals, thimbles containing 1 g of sample were attached to Soxhlet beakers containing sufficient petroleum ether to submerge the sample. Samples were defatted via Soxhlet extraction, and the defatted samples and Soxhlet beakers were dried overnight in air ovens at 105 °C. The mass of fat was quantified by taking the masses of the dried Soxhlet beakers after cooling (*M*_fat_).

The total available carbohydrates and dietary fibre of test meals were determined according to the procedures described in the commercially available Available Carbohydrates and Dietary Fibre Assay kit (K-ACHDF, Megazyme, Wicklow, Ireland), which are based on the methods by Lee, et al.^55^ and Prosky, et al.^56^. Briefly, 1 g of defatted sample in 40 mL of MES-TRIS buffer was incubated at 80 °C with 50 μL of thermostable α-amylase (3000 U/mL) for 35 min, then at 60 °C with 100 μL of protease (350 U/mL) for 30 min, and lastly at 60 °C with 200 μL of amyloglucosidase (3300 U/mL) for 30 min after a pH adjustment to 4.1–4.5. The mixture was then diluted 50-fold with sodium maleate buffer. Ethanol (95% v/v) was added to the mixture and allowed to stand for 60 min for the precipitation of soluble dietary fibre. The residue was subsequently separated from the suspension via suction filtration through crucibles, dried overnight at 105 °C in an air oven, and weighed (*M*_fibre_).

Dried residues were used for protein analysis using the Kjeldahl method with *N* × 6.25 as the protein conversion factor to calculate the mass of protein (*M*_protein_), and ash analysis by incinerating the residue at 525 °C for 5 h and weighing the resultant ash (*M*_ash_).

The distribution of proximates were determined using Eqs. (1) to (5). The proximate contents of test meals are as presented in Supplemental Table 1.

1 $${Fat}\,\left( \% \right)=\frac{{M}_{{fat}}}{{M}_{{sample}}}\times 100$$

2 $${Dietary}\,{fibre}\,\left( \% \right)=\frac{{M}_{{fibre}}-{M}_{{protein}}-{M}_{{ash}}}{{M}_{{sample}}}\times 100$$

3 $${Protein}\,\left( \% \right)=\frac{{M}_{{protein}}}{{M}_{{sample}}}\times 100$$

4 $${Ash}\,\left( \% \right)=\frac{{M}_{{ash}}}{{M}_{{sample}}}\times 100$$

5 $${Available}\,{carbohydrates}\,\left( \% \right)=100-{Moisture}\,\left( \% \right)-{Fat}\left( \% \right)-{Dietary}\,{fibre}\,\left( \% \right)-{Protein}\left( \% \right)-{Ash}( \% )$$

### Determination of anthocyanin content

Anthocyanin extraction was performed according to the methods by Ou, et al.^14^. Briefly, BRAE (1.0 g) and lyophilized bread powders (5.0 g) were weighed into 50 mL centrifuge tubes and macerated with 10 mL of 0.01% v/v trifluoroacetic acid in methanol for 30 min using an orbital shaker at 200 rpm. The tubes were centrifuged at 8000 × *g* at 20 °C for 10 min before collecting the liquid fraction. Maceration of the solid fractions were repeated four times. The combined liquid fractions were filtered through Whatman^®^ qualitative filter paper (Grade 1, Z274852), before being condensed by evaporating methanol at 40 °C under vacuum. The condensed liquid extracts were made up to 5 mL with 0.1 % v/v formic acid and stored in the dark at −20 °C until analysis. The final liquid extracts from both BRAE and lyophilized bread powders were diluted 200- and 10-times respectively, before filtering it using a 0.22 μm PTFE filter for analysis.

The pH differential method for total monomeric anthocyanin is carried out in accordance to the AOAC Official Method 2005.02^57^. Briefly, 20-times dilutions of final liquid extracts were prepared in pH 1.0 (potassium chloride, 0.025 M) and pH 4.5 (sodium acetate, 0.4 M) buffers. This dilution factor was previously determined by diluting the test sample with pH 1.0 buffer until its absorbance at 520 nm was between 0.2 and 1.4 AU. The test samples were read against a blank cell of deionized water at 520 and 700 nm, within 50 min of preparation. The total monomeric anthocyanin content (TAC) was expressed in C3G equivalents according to Eq. (6).

6 $${TAC}=\{[{\left({A}_{520{nm}}-{A}_{700{nm}}\right)}_{{pH}1.0}-{\left({A}_{520{nm}}-{A}_{700{nm}}\right)}_{{pH}4.5}]\times {MW}\times {DF}\times {10}^{3}\}/(\varepsilon \times 1)$$

where molecular weight, MW = 449.2 g/mol for C3G; dilution factor, DF = 20, molar extinction coefficient, *ɛ* = 26900 L cm^−1^ mol^−1^ for C3G.

To determine the distribution of major ACN in BRAE and BB, extracts were analyzed using a reverse-phased C_18_ Sunfire column (250 × 4.6 mm/5 μm; Waters, Wexford, Ireland) on an HPLC system (Shimadzu Prominence, Shimadzu, Kyoto, Japan) connected with a diode array detector (DAD). The injection volume was 50 μL. The flow rate and oven temperature were maintained at 1 mL/min at 25 °C. A gradient elution process was applied (mobile phase A: 5% v/v formic acid; mobile phase B: 100% acetonitrile): 0% B for 5 min, 10% B at 20 min, 13% B at 40 min, 20% B at 44 min, 25% B at 50 min, and 100% B at 55 min. Detection of ACN was performed at 520 nm. Identification and quantification of each anthocyanin was based on matching the retention time and peak areas with the external calibration curve of respective standards. The anthocyanin content of test meals is as presented in Supplemental Table 1.

### In-vitro quantification of starch hydrolase inhibition

To determine the inhibitor activities of major ACN species, 50 μL of enzyme solution was mixed with 50 μL of inhibitor solution of different concentrations and incubated for 15 min at 37 °C. The reaction was initiated by mixing 100 μL of 1% gelatinized wheat starch solution into the enzyme-inhibitor mixtures. Changes in turbidity were immediately monitored at 660 nm for 2 h in a microplate reader preincubated at 37 °C, with one reading per minute, while shaking in between readings to minimize starch sedimentation. Enzyme activities were inversely proportional to the AUC for turbidity readings. The percentage inhibition (*%I*) was defined as:

7 $$\% I=({{AUC}}_{{Sample}}-{{AUC}}_{{Control}})/{{AUC}}_{{Sample}}\times 100$$

in which ${{AUC}}_{{Sample}}$ is the area under the curve for the inhibitor, and ${{AUC}}_{{Control}}$ is the area under the curve without inhibitors.

### In-vitro quantification of pancreatic lipase inhibition

The inhibitory effects of 4-BBgr on pancreatic lipase was assessed using 4-methylumbelliferyl oleate (4-MUO) as a substrate. Equal volumes of 0.1 mM 4-MUO and intestinal digesta (pH 7.0) containing pancreatic lipase (2000 U/mL final concentration) were mixed to initiate the enzymatic reaction. The amount of 4-methylumbelliferone released by lipase was measured over at 0, 2, 4, 8, 15, 30, 45, 60, 90, 120, 180, and 240 min using a fluorescence microplate reader (Varioskan Flash, Thermo Scientific, MA, USA). The excitation and emission wavelengths were set at 320 nm and 450 nm respectively. As an indication of overall lipase activity, the AUC was determined from the hydrolysis curve of 4-BBgr using the trapezoid rule and compared to that of CONBgr. The percentage inhibition (*%I*) was defined as:

8 $$\% I=({{AUC}}_{{Sample}}-{{AUC}}_{{Control}})/{{AUC}}_{{Sample}}\times 100$$

in which ${{AUC}}_{{Sample}}$ is the area under the curve for the inhibitor, and ${{AUC}}_{{Control}}$ is the area under the curve without inhibitors.

### Simulated gastrointestinal digestion of test meals

The starch digestion profiles of 2-, 4-BB, and 4-BBgr, relative their respective controls (CON and CONBgr), were evaluated in-vitro using a simulated gastrointestinal digestion model^14^. To simulate the oral phase, fresh meal challenges were briefly homogenized in distilled water (1:5 w/v) to simulate the mastication process. Simulated salivary fluid (SSF), 0.3 M CaCl_2_ (1.5 mM final concentration), and α-amylase prepared in SSF (75 U/mL final concentration) were added to 2 g of homogenized samples. The mixture was topped up to volume with distilled water, and vortexed briefly before incubation in a 37 °C water bath for 2 min with agitation.

To simulate gastric digestion, simulated gastric fluids (SGF), 0.3 M CaCl_2_ (0.15 mM final concentration), and pepsin prepared in SGF (2000 U/mL final concentration) were added to oral mixtures. The pH of resulting gastric mixtures was adjusted to 3.0 with 6 M HCl, topped up to volume with distilled water, and vortexed briefly before incubation in a 37 °C water bath for 2 h with agitation.

To simulate intestinal digestion, simulated intestinal fluids (SIF), 0.3 M CaCl_2_ (0.6 mM final concentration), pancreatin prepared in SIF (100 U/mL final concentration based on trypsin activity), and α-amyloglucosidase solution (1.3 U/mL final concentration) were added to gastric mixtures. Additional lipase prepared in SIF (2000 U/mL final concentration) was added for the digestion of HC/HF meal samples. The pH of resulting intestinal mixtures were adjusted to 7.0 with 6 M NaOH, topped up to volume with distilled water, and vortexed briefly before incubation in a 37 °C water bath for 4 h with agitation. Aliquots of 100 μL were withdrawn at 0, 15, 30, 45, 60, 75, 90, 120, 150, 180, 210, and 240 min; and thereafter added to 900 μL of ethanol to quench the enzyme activity. The preparation of simulated digestion fluids and required reagents for each digestion phase are provided in Supplemental Table 2A, B.

Quenched aliquots were centrifuged at 15,000 × *g* for 10 min, and the supernatant was used for the quantification of glucose released according to the methods stated in the Megazyme D-glucose hexokinase assay kit (K-GLUHK, Megazyme, Wicklow, Ireland). Digestibility data was fitted into the first order equation Eq. (9).

9 $${C}_{t}={C}_{0}+\left({C}_{\infty }-{C}_{0}\right)\left(1-{e}^{-{kt}}\right)$$

where ${C}_{t}$ is the amount of glucose released at time $t$ (minutes), ${C}_{0}$ is the amount of glucose released at $t=0$ min, ${C}_{\infty }$ is the equilibrium percentage of glucose released after 240 min, and $k$ is the kinetic constant. Using the fitted hydrolysis curves obtained from Eq. (9), the IAUC was determined using the trapezoid rule.

### Blood collection and biochemical measurements

Phlebotomy was carried out on the arm not used for blood pressure measurements, using a butterfly cannula. Blood samples for plasma glucose measurements were collected in sodium fluoride tubes and were inverted 6–8 times post collection. Blood samples for serum insulin and lipid panel measurements were collected in plain tubes and left to clot for 30 min. Blood samples for bioavailability analysis were collected in EDTA tubes and inverted 6–8 times after collection. Plasma was then separated from these samples by centrifugation at 3500 × *g* for 15 min.

Blood samples for glucose, insulin, and lipid panel were sent to Quest Laboratories, Singapore, for biochemical analysis. Plasma glucose was determined spectrophotometrically by the hexokinase/glucose-6-phosphate dehydrogenase method. Serum insulin was determined by the electrochemiluminescence immunoassay method. Serum triglycerides (TG) were determined by colorimetry using the Fossati 3-step enzymatic reaction involving lipase, glycerol kinase, and glycerol-3-phosphate-oxidase, with a Trinder endpoint reaction. Total cholesterol (TC) was determined by colorimetry using the cholesterol esterase/cholesterol oxidase method with a Trinder endpoint reaction. High-density lipoprotein (HDL) cholesterol was determined by the Trinder colorimetric method. LDL cholesterol was calculated using the Friedewald equation.

### GI determination

Blood glucose measurements from the *Bread* trial were used for the determination of GI in accordance with ISO 26642:2010^52^. Time-series blood glucose curves of the 50-g glucose standard, CON, 2-BB, and 4-BB were constructed to obtain glucose IAUCs via the trapezoid rule. The GI of CON, 2-BB, and 4-BB were determined by dividing their respective IAUCs by that of the glucose standard. The GI of each bread was expressed as the mean and standard deviation from the respective average GIs of 24 participants.

### Bioavailability determination of anthocyanins

Bioavailability analysis was performed in the *Bread* trial according to the methods by Kay, et al.^58^. Plasma ACNs and their metabolites were extracted using C_18_ solid-phase extraction (SPE) cartridges (400020, Cayman Chemical, Michigan, USA) that had been preconditioned with 7 mL acidified methanol (0.1% v/v trifluoroacetic acid, pH 2.1) and then with 7 mL acidified water (10 mM oxalic acid, pH 2.2). Blood plasma was acidified with 40 μL 6 M HCl, diluted in an equal volume of 10 mM oxalic acid, vortexed, and loaded directly into the SPE cartridge. The sample was drained off in a vacuum manifold and washed with 2 volumes of acidified water. The remaining ACN extract was eluted with 6 mL acidified methanol, and subsequently brought to dryness at 25 °C under a continuous stream of nitrogen gas. The residue was dissolved in 200 μL of 0.1% v/v formic acid and filtered through a 13 mm 0.22 μm PTFE hydrophilic syringe filter. The resulting solution was analyzed using a reverse-phased C_18_ Sunfire column (250 × 4.6 mm/5 μm; Waters, Wexford, Ireland) on an HPLC system (Shimadzu Prominence, Shimadzu, Kyoto, Japan) connected with a DAD. The injection volume was 50 μL. The flow rate and oven temperature were maintained at 1 mL/min at 25 °C. A gradient elution process was applied (mobile phase A: 5% v/v formic acid; mobile phase B: 100% acetonitrile): 0% B for 5 min, 10% B at 20 min, 13% B at 40 min, 20% B at 44 min, 25% B at 50 min, and 100% B at 55 min. Detection of ACNs was performed at 520 nm; while its metabolites, ferulic acid, vanillic acid, and protocatechuic acid were detected at 280 nm. Identification and quantification of each ACN and metabolite were based on matching the retention time and peak areas with the external calibration curve of respective standards.

### Quantification of lipoprotein particles and subfractions

Lipoprotein measurements were quantified in the *Burger* trial by the Nightingale high-throughput nuclear magnetic resonance (NMR) platform (Nightingale Health, Helsinki, Finland) according to previously described methods^59,60^. The investigators at Nightingale Health were blinded prior to the sample analysis, which was performed in one batch to eliminate batch effects. The biomarkers measured included apolipoprotein concentrations, lipoprotein particle sizes, as well as plasma concentrations of total and subfractions of lipoproteins. The 14 lipoprotein subclasses assessed comprised extremely large very low-density lipoproteins (XXL-VLDL), very large very low-density lipoproteins (XL-VLDL), large very low-density lipoproteins (L-VLDL), medium very low-density lipoproteins (M-VLDL), small very low-density lipoproteins (S-VLDL), very small very low-density lipoproteins (XS-VLDL), intermediate-density lipoprotein (IDL), large low-density lipoprotein (L-LDL), medium low-density lipoprotein (M-LDL), small low-density lipoprotein (S-LDL), very large high-density lipoprotein (XL-HDL), large high-density lipoprotein (L-HDL), medium high-density lipoprotein (M-HDL), and small high-density lipoprotein (S-HDL). It was not possible to distinguish chylomicrons (CM) and their remnants from VLDL through NMR spectroscopy, hence they were quantified together with the VLDL fractions^61^. It was reported that while CM are part of the XXL-VLDL subfractions, CM remnants can be included in any VLDL subfraction, contingent to their particle sizes.

### Statistical analysis

Power calculation for the *Bread* trial was based on a hypothesized 1.6 mmol/L decrease in the maximal postprandial glucose concentration with blueberry supplementation^62^. With an 80% power and 95% confidence level, the crossover trial was designed to include a minimum of 15 participants to observe a significant difference in incremental glucose. On the other hand, power calculation for the *Burger* trial was based on blood glucose responses reported by Schell et al.^63^, in which acute ACN-rich raspberry supplementation with a high-fat meal resulted in a 1.9 mmol/L decrease in maximal postprandial blood glucose concentrations with a standard deviation of 1.5 mmol/L. With an 80% power and 95% confidence level, this study was designed to include a minimum of 12 participants to observe a significant attenuation in glucose concentrations. However, there was no available data on the effects on black rice ACNs in similar study designs during power calculation. While blueberries and raspberries were classified as ACN-rich whole foods, BB were considered as foods fortified with an ACN-rich food extract. Due to the inherent differences between the test foods referenced, and to account for a 20% dropout rate, a larger sample size of 24 participants was recruited for each study.

Statistical analysis was performed using GraphPad Prism 9.0 (GraphPad, San Diego, CA, USA), with statistical significance determined at *p* < 0.05. Statistical differences in simulated starch digestibility measurements among CON, 2-BB, 4-BB were determined by one-way analysis of variance with Tukey’s multiple comparison test. Unpaired *t* tests were used to compute the differences in in-vitro starch digestibility measurements and lipase activities between CONBgr and 4-BBgr. Repeated measures one-way ANOVA with Tukey’s multiple comparison test was used to compare the effects of BRAE fortification on the changes in GI, and postprandial incremental blood measurements of glucose and insulin for the *Bread* trial. Repeated measures two-way ANOVA with Bonferroni’s multiple comparison test were used to compare the postprandial differences in polyphenolic bioavailability between 2-BB and 4-BB at 60 and 120 min. Paired *t* tests were used to assess the postprandial changes in glucose, insulin, lipid panel, and lipoproteins to the ACN-fortified HC/HF-meal challenges for the *Burger* trial. In a secondary analysis, two-way ANOVA was also performed to analyze the effects of BRAE fortification on postprandial glycemic and lipidemic responses in both *Bread* and *Burger* trials, with the intervention group and time points identified as fixed factors.

### Reporting summary

Further information on research design is available in the Nature Research Reporting Summary linked to this article.

### Supplementary information

Supplementary Data

Reporting summary

## Data Availability

Data described in the manuscript will be made available on request to the corresponding author.
