# Supplementary material for: Postprandial glycemic and lipidemic effects of black rice anthocyanin extract fortification in foods of varying macronutrient compositions and matrices
Source: NPJ Sci Food. 2023 Nov 1;7:59. doi: 10.1038/s41538-023-00233-y (PMC10620212; doi:10.1038/s41538-023-00233-y)
Supplement: Supplementary file 1 — Supplementary Data [file 41538_2023_233_MOESM1_ESM.pdf]

Online Supporting Material 1 - In-vitro characterization of test meals

SUPPLEMENTAL TABLE 1

Proximate analysis and total anthocyanin content of test meals and meal components

	ACHO (g)	Protein (g)	Fat (g)	TAC	C3G	P3G	C3R
HC test meals	g/serve			mg/serve			
CON	50	7.8	2.5	0	n.d	n.d	n.d
2-BB	50	8.1	2.4	60.3	44.3	6.2	9.8
4-BB	50	8.3	2.5	127.3	93.6	12.9	20.8
HF-test meals							
Beef patty	< 1	25.5	36.8	0	n.d	n.d	n.d
Mayonnaise	< 1	< 1	4.5	0	n.d	n.d	n.d
CONgr	50	33.3	43.8	0	n.d	n.d	n.d
4-BBgr	50	33.8	43.8	127.3	93.6	12.9	20.8
	ACHO	Protein	Fat				
	kcal (% total kcal)			Total kcal			
CONgr	200 (27.5%)	133.2 (18.3%)	394.2 (54.2%)	727.4			
4-BBgr	200 (27.4%)	135.2 (18.5%)	394.2 (54.0%)	729.4			

n.d., not determined; C3G, cyanidin-3-glucoside; P3G, peonidin-3-glucoside; C3R, cyanidin-3-rutinoside; TAC, total anthocyanin content. C3G, P3G, and C3R were determined by HPLC analysis. Total phenolic content is expressed as gallic acid equivalents. Total anthocyanin content is determined from the total peak area of quantified anthocyanins from HPLC analysis.

Online Supporting Material 2 – Reagents used in the simulated gastrointestinal digestion

SUPPLEMENTAL TABLE 2A

Composition of simulated digestion fluids (SF).

For 1000mL of each of SF								
			**SSF (pH 7)**		**SGF (pH 3)**		**SIF (pH 7)**	
**Stock solution**	**Stock Concentration**		**Volume of stock added**	**Final concentration**	**Volume of stock added**	**Final concentration**	**Volume of stock added**	**Final concentration**
	**(g/L)**	**(M)**	**(mL)**	**(mM)**	**(mL)**	**(mM)**	**(mL)**	**(mM)**
KCl	37.3	0.5	37.75	15.1	17.25	6.9	17	6.8
KH_2PO_4	68	0.5	9.25	3.7	2.25	0.9	2	0.8
$NaHCO_3$	84	1	17	13.6	31.25	25	106.25	85
NaCl	117	2	0	0	29.5	47.2	24	38.4
$MgCl_2(H_2O)_6$	30.5	0.15	1.25	0.15	1	0.12	2.75	0.33
$(NH4)_2CO_3$	48	0.5	0.15	0.06	1.25	0.5	0	0
$CaCl_2(H_2O)_2$	44.1	0.3	0.0625	1.5	0.0125	0.15	0.1	0.6
HCl		6	0.225	1.1	3.25	15.6	1.75	8.4

SSF, simulated salivary fluid; SGF, simulated gastric fluid; SIF, simulated intestinal fluid.

SUPPLEMENTAL TABLE 2B

Usage of reagents in each phase of the simulated gastrointestinal digestion.

	**Oral phase (pH 7)**	**Gastric phase (pH 3)**		**Intestinal phase (pH 7)**
Sample (g)	2	4		8
Simulation fluid (μL)	1300	2950		4510
0.3M $CaCl_2$ (μL)	10	2		16
Enzyme	α-amylase from porcine pancreas	Pepsin from porcine pancreas	Pancreatin from porcine pancreas	α-amyloglucosidase from *Aspergillus niger*
Final enzyme activity (U/ mL)	75	2000	100	1.3
Volume of 6M HCl/ NaOH	Added to adjust to pH 7	Added to adjust to pH 3		Added to adjust to pH 7
Volume of water	Top up to total volume	Top up to total volume		Top up to total volume
Total volume (mL)	4	8		16

Online Supporting Material 3 – Inhibition of *in-vitro* starch hydrolase activity

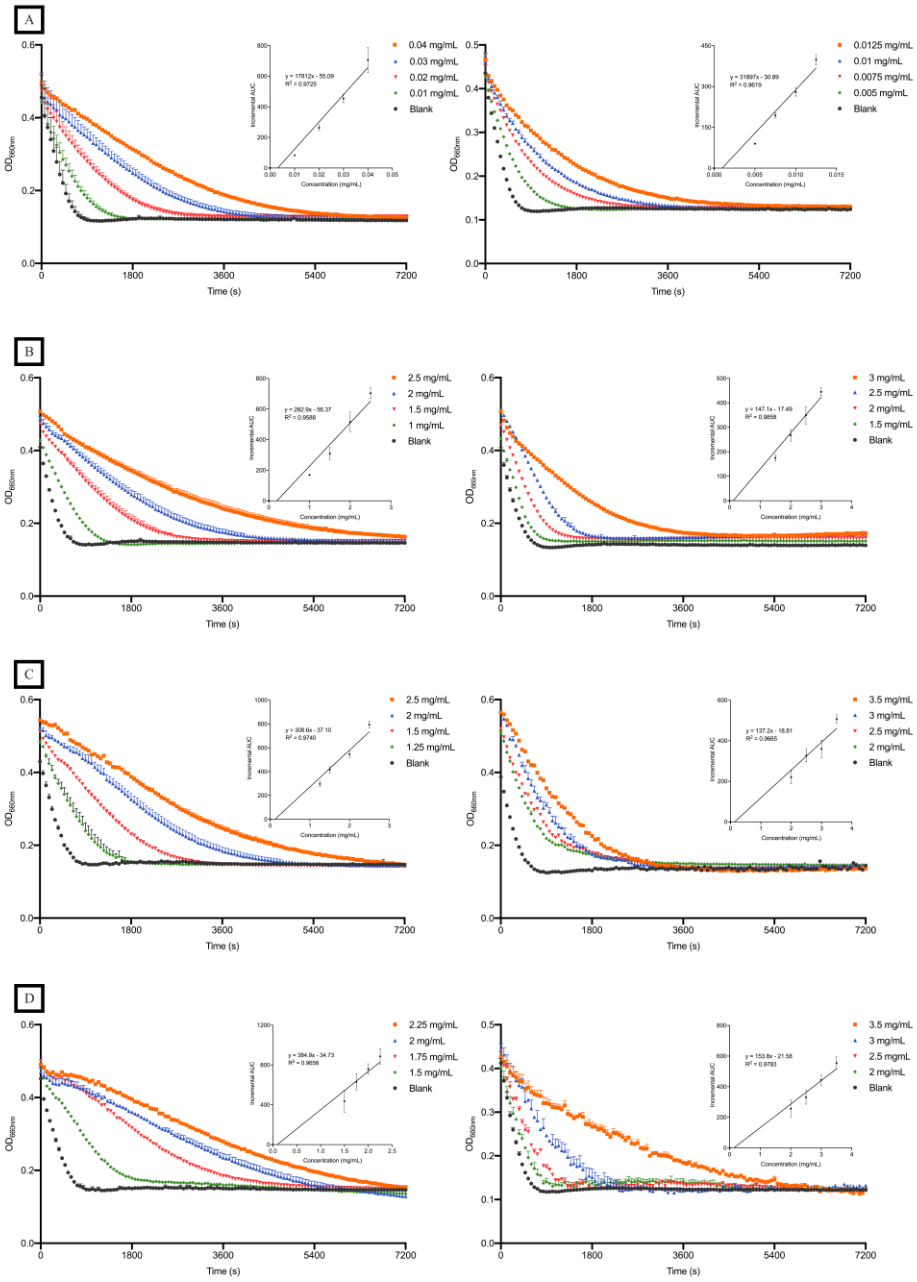

SUPPLEMENTAL FIGURE 3

Representative kinetic curves for starch hydrolysis with varying concentrations of acarbose and major black rice anthocyanins, as assessed by changes in starch turbidity. Insets: dose-response relationships of incremental AUC against inhibitor concentrations. Left: turbidity changes in α-amylase; right: turbidity changes in α-glucosidase. **A:** acarbose; **B:** cyanidin-3-glucoside; **C:** peonidin-3-glucoside; **D:** cyanidin-3-rutinoside. Values are represented as means and standard error of means.

Online Supporting Material 4 – Simulated gastrointestinal digestion of BRAE-fortified, single HC food

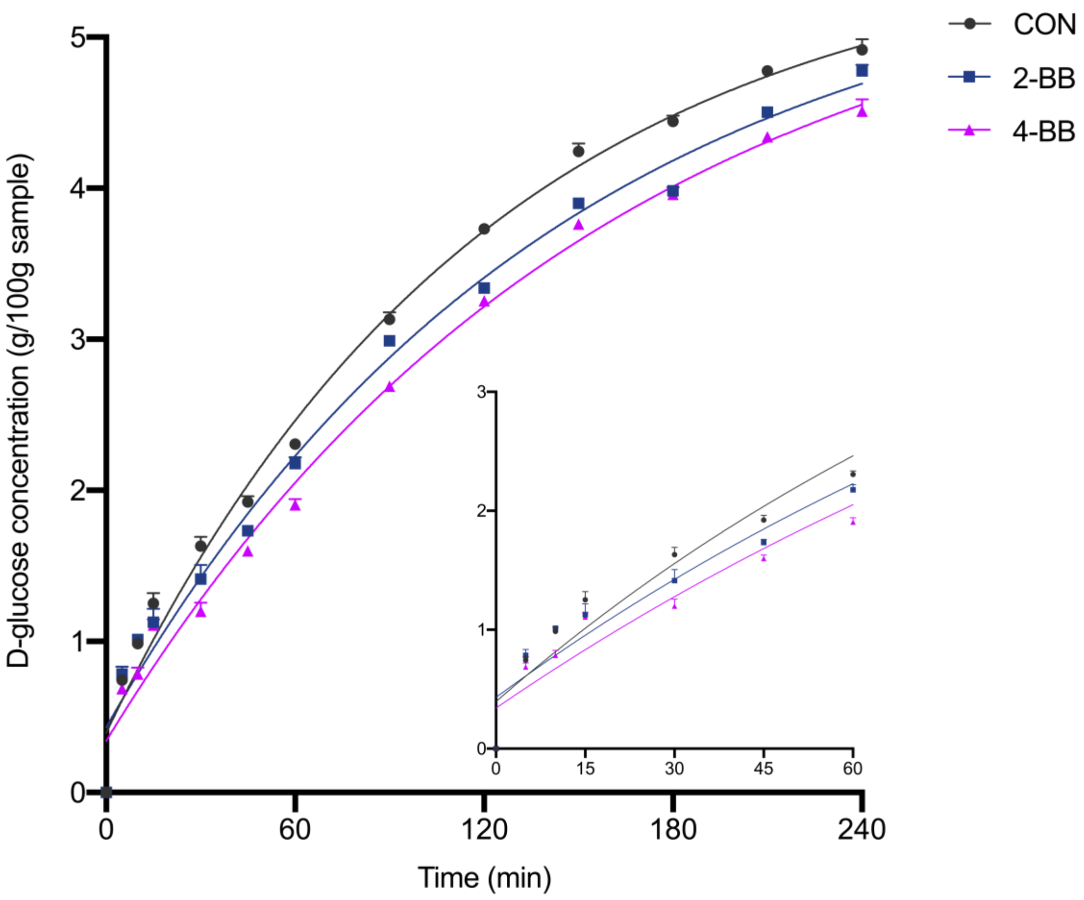

SUPPLEMENTAL FIGURE 4

Inhibitory effects of BRAE enrichment at 0% (CON), 2% (2-BB), and 4% (4-BB) levels in wheat bread on starch digestibility, using a simulated digestion model. Insets: starch hydrolysis curves in the first 60 min of simulated starch digestion. Values are represented as means and standard error of means.

Online Supporting Material 5 – Postprandial glycemic responses to a BRAE-fortified, single HC-food

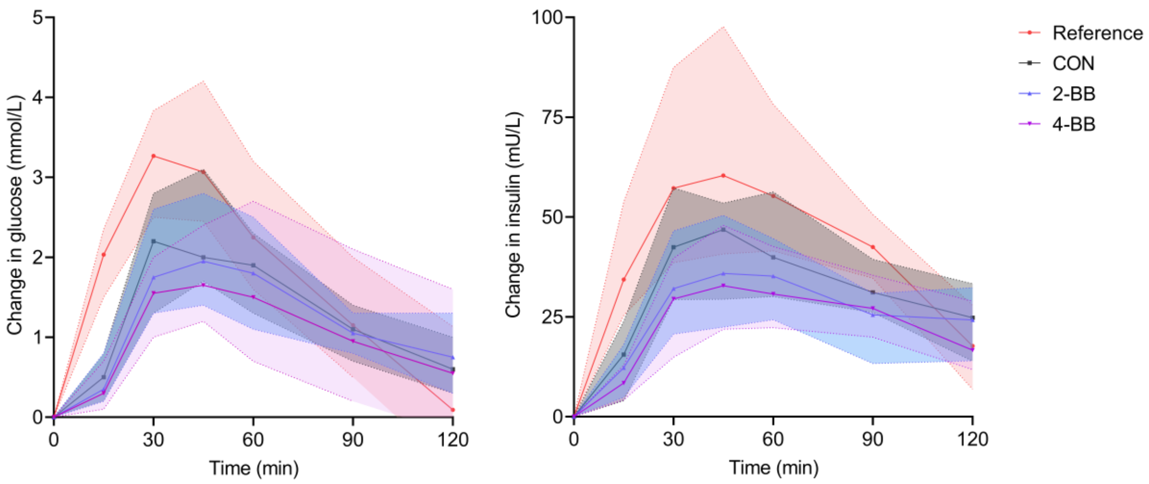

SUPPLEMENTAL FIGURE 5

Postprandial glycemic responses to the 50-g glucose beverage, and 50-g ACHO equivalent HC test meals in the *Bread* (n = 22) trial. **Left:** incremental change in glucose over 2 h; **Right:** incremental change in insulin over 2 h. Values are represented as medians and 95% confidence intervals.

SUPPLEMENTAL TABLE 5

Two-way ANOVA summary table for the changes in postprandial glycemia in response to the 50-g ACHO equivalent HC test meals in the *Bread* (n = 22) trial.

			**Incremental glucose (mmol/L)**		
	SS	***df***	**MS**	***F***	***p***
Intervention	4.04	2	2.02	F (1.620, 34.01) = 1.87	0.175
Time (min)	213.2	6	35.54	F (2.329, 48.90) = 37.81	**<0.0001**
Time × Intervention	4.91	12	0.41	F (4.529, 95.11) = 1.45	0.218
Subject (matching)	113.8	21	5.42		
Subject × Intervention	45.26	42	1.08		
Subject × Time	118.4	126	0.94		
Residual	113.8	252	0.28		
			**Incremental insulin (mU/L)**		

	SS	***df***	**MS**	***F***	***p***
Intervention	5046	2	2523	F (1.621, 34.05) = 2.10	0.146
Time (min)	93740	6	15623	F (2.511, 52.74) = 37.52	**<0.0001**
Time × Intervention	3025	12	252.1	F (3.461, 72.68) = 1.28	0.288
Subject (matching)	66007	21	3143		
Subject × Intervention	50521	42	1203		
Subject × Time	52464	126	416.4		
Residual	49698	252	197.2		

SS: sum of squares; df: degree of freedom; MS: mean square; *p*: *p*-value for the observed differences between CON, 2-BB, and 4-BB groups analysed with a repeated measures two-way ANOVA and Bonferroni's multiple comparisons test. Statistical significance was determined at $p < 0.05$ and is represented in bold.

Online Supporting Material 6 – Postprandial bioavailability of major black rice anthocyanins and metabolites

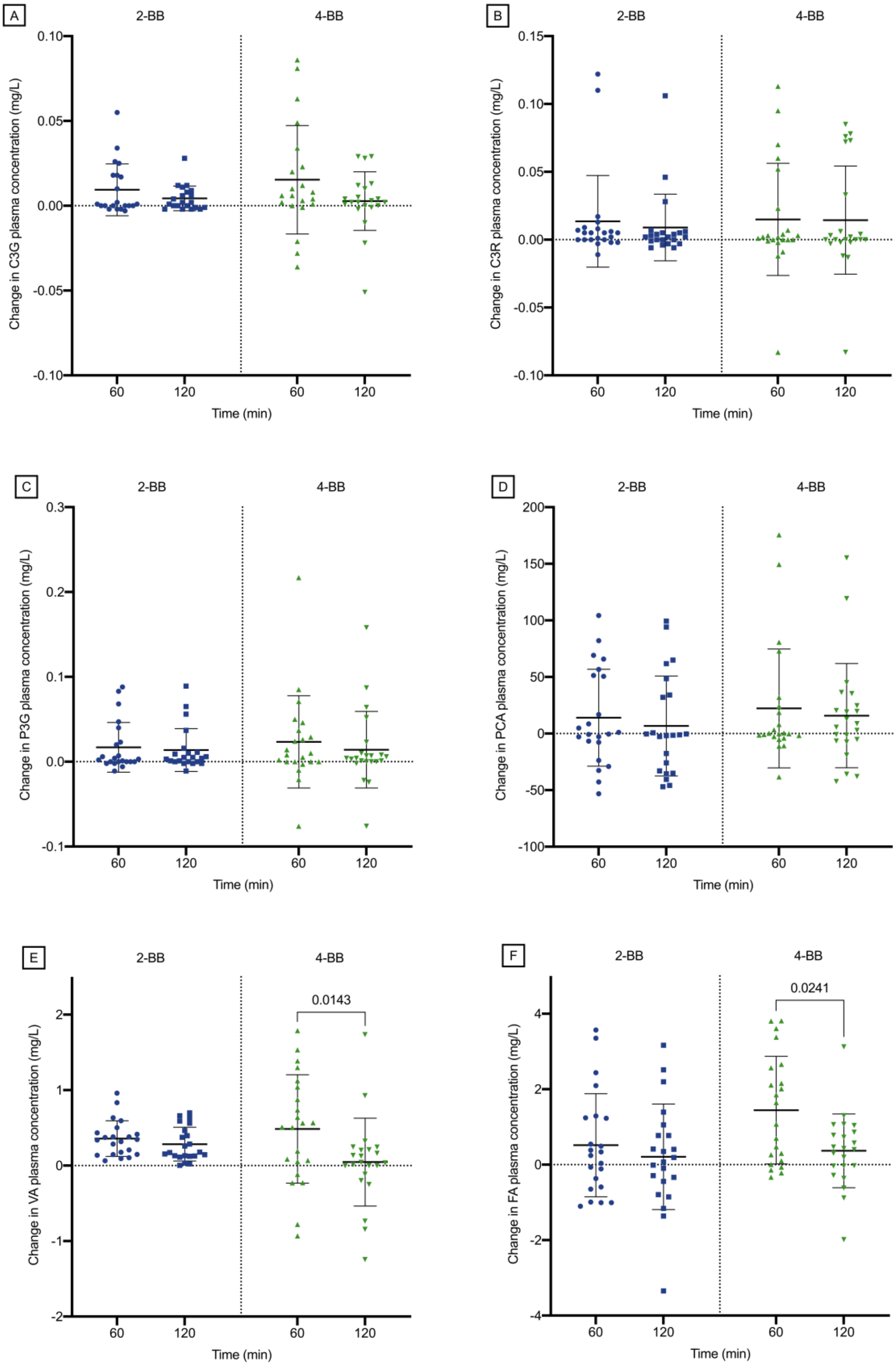

SUPPLEMENTAL FIGURE 6.

Changes in postprandial plasma concentrations at 60 and 120 min from baseline in major black rice anthocyanins (**A – C**) and phenolic metabolites (**D – F**). **A:** C3G; **B:** C3R; **C:** P3G; **D:** PCA; **E:** VA; **F:** FA. Differences among digestion phases and between experimental models were determined by a repeated measures two-way ANOVA with Bonferroni's multiple comparisons test. Values are represented as means and 95% confidence intervals. Each data point represents the plasma concentration for each participant (n = 22).

Online Supporting Material 7 – Simulated gastrointestinal digestion of a BRAE-fortified, HC/HF mixed-nutrient meal

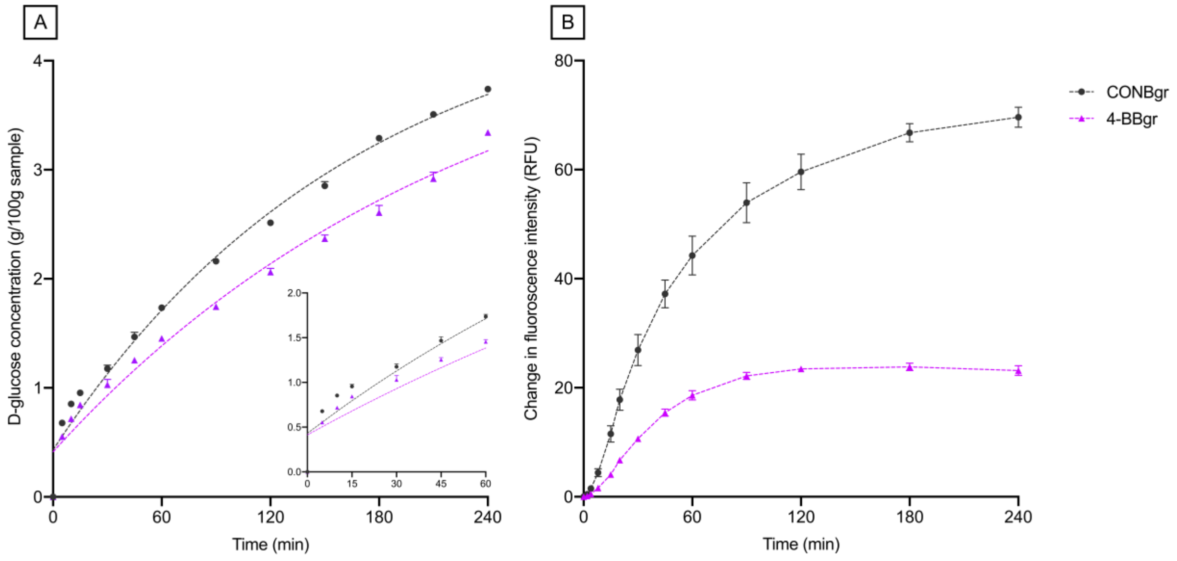

SUPPLEMENTAL FIGURE 7

Inhibitory effects of BRAE enrichment on starch digestibility and lipase activity. Insets: starch hydrolysis curves in the first 60 min of simulated starch digestion. A: simulated starch digestibility of the HC/HF-meal challenge with (4-BBgr) and without BRAE (CONBgr); B: inhibitory effects of BRAE on pancreatic lipase, determined through the simulated digestion of CONBgr and 4-BBgr. Values are represented as means and standard error of means.

Online Supporting Material 8 – Postprandial glycemic and lipidemic responses to a BRAE-fortified, HC/HF mixed-nutrient meal

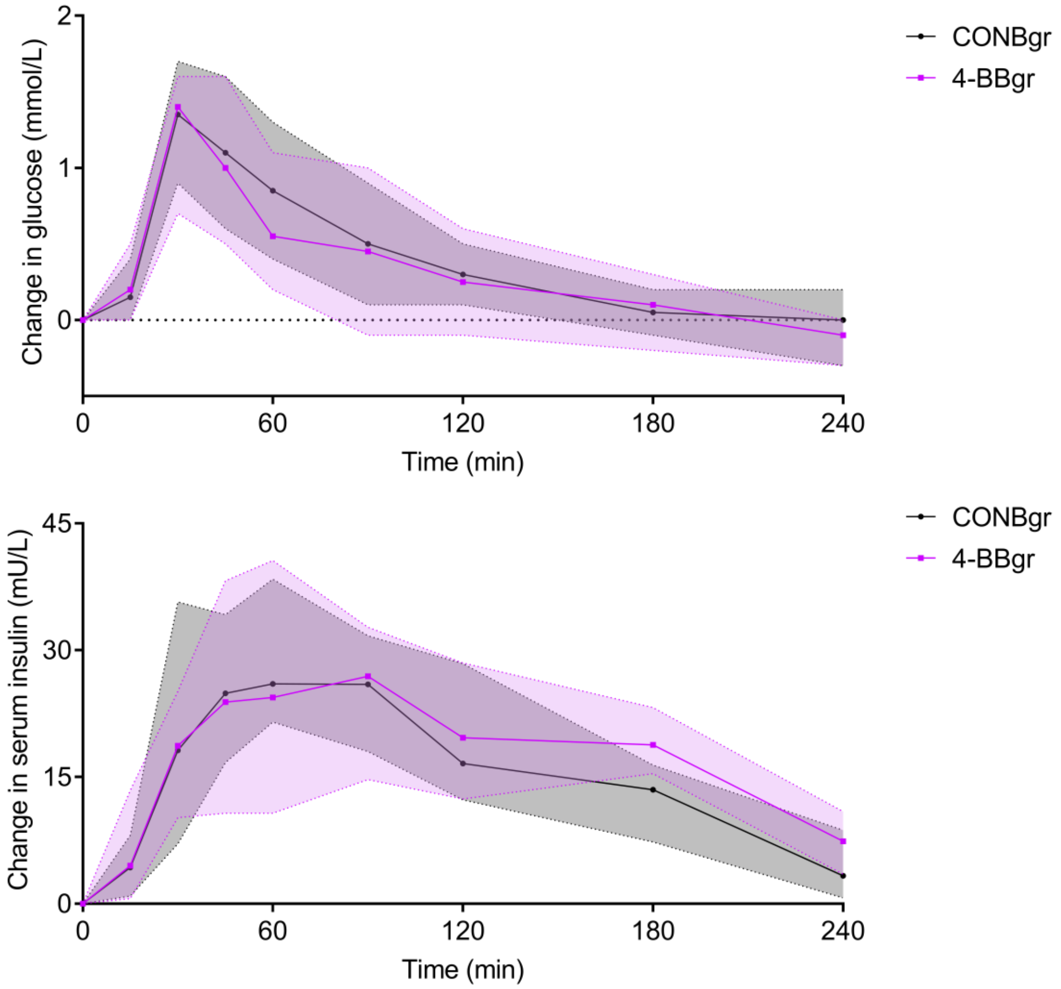

SUPPLEMENTAL FIGURE 8A

Postprandial glycemic responses to the 50-g ACHO equivalent HC/HF mixed meal challenge in the *Burger* (n = 24) trial. **Top:** incremental change in glucose over 4 h; **Bottom:** incremental change in insulin over 4 h. Values are represented as medians and 95% confidence intervals.

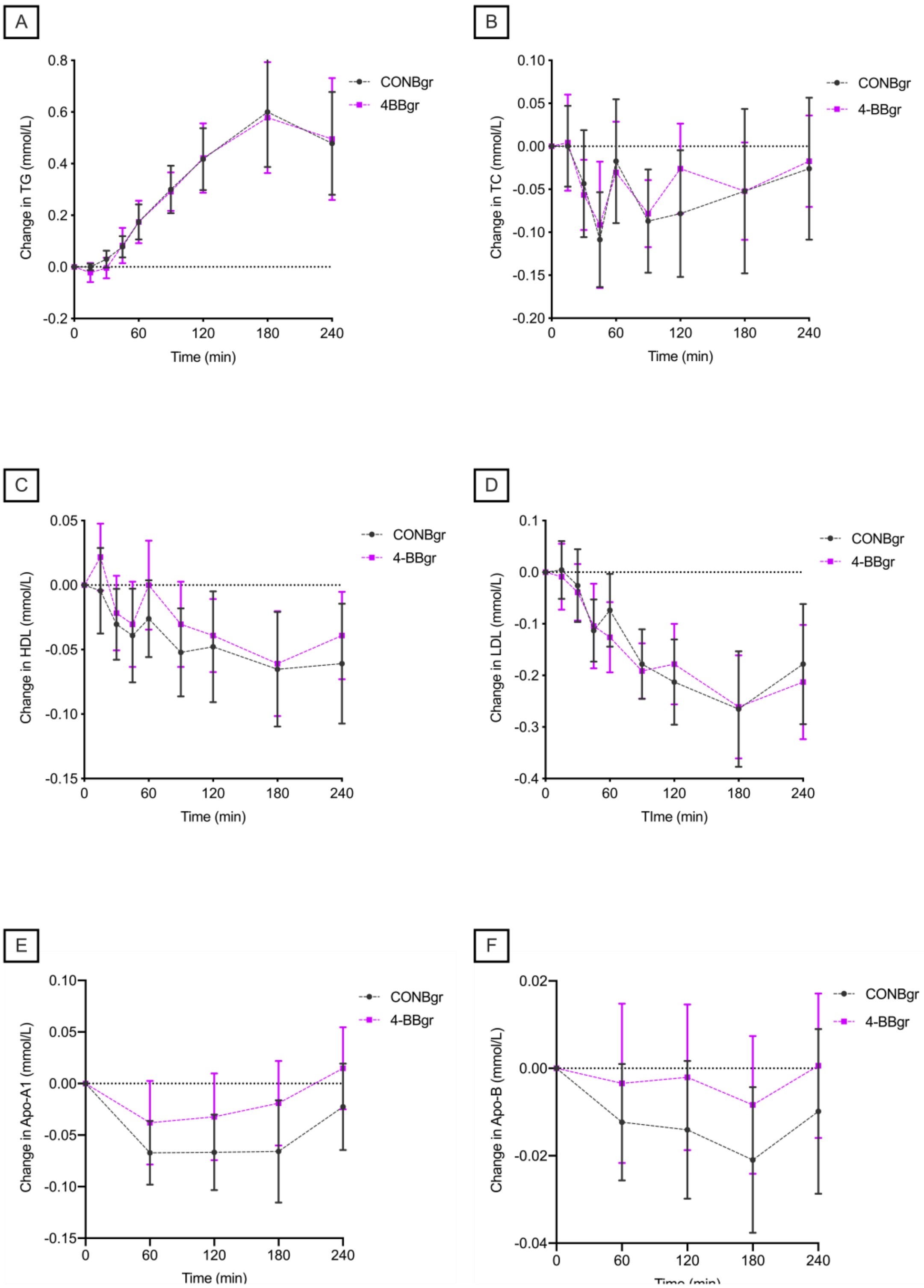

SUPPLEMENTAL FIGURE 8B

Postprandial lipidemic responses over 4 h to 50-g ACHO equivalent HC/HF test meals in the *Burger* trial (n = 24). **A:** incremental change in TG; **B:** incremental change in TC; **C:** incremental change in HDL-c; **D:**

incremental change in LDL-c; **E:** incremental change in Apo-A1; **F:** incremental change in Apo-B. Values are represented as means and 95% confidence intervals.

SUPPLEMENTAL TABLE 8

Two-way ANOVA summary table for the changes in postprandial glycemia and lipidemia in response to the 50-g ACHO equivalent HC/HF test meals in the *Burger* (n = 24) trial.

**Incremental glucose (mmol/L)**					
	SS	***df***	**MS**	***F***	***p***
Intervention	0.37	1	0.37	F (1, 23) = 0.55	0.466
Time (min)	93.07	8	11.63	F (8, 184) = 33.32	**<0.0001**
Time × Intervention	0.96	8	0.12	F (8, 184) = 0.80	0.599
Subject (matching)	40.16	23	1.75		
Subject × Intervention	15.60	23	0.68		
Subject × Time	64.24	184	0.35		
Residual	27.48	184	0.15		
**Incremental insulin (mU/L)**					
	SS	***df***	**MS**	***F***	***p***
Intervention	75.08	1	75.08	F (1, 23) = 0.12	0.735
Time (min)	46281	8	5785	F (8, 184) = 22.13	**<0.0001**
Time × Intervention	1724	8	215.5	F (8, 184) = 1.08	0.378
Subject (matching)	25639	23	1115		
Subject × Intervention	14653	23	637.1		
Subject × Time	48097	184	261.4		
Residual	36658	184	199.2		
**Incremental TG (mmol/L)**					
	SS	***df***	**MS**	***F***	***p***
Intervention	0.0047	1	0.0047	F (1, 22) = 0.083	0.776
Time (min)	19.26	8	2.41	F (8, 176) = 27.94	**<0.0001**
Time × Intervention	0.025	8	0.0031	F (8, 176) = 0.266	0.976
Subject (matching)	11.56	22	0.526		
Subject × Intervention	1.26	22	0.057		
Subject × Time	15.16	176	0.086		
Residual	2.06	176	0.012		
**Incremental TC (mmol/L)**					

	SS	*df*	MS	*F*	*p*
Intervention	0.0054	1	0.0054	F (1, 22) = 0.11	0.741
Time (min)	0.45	8	0.056	F (8, 176) = 4.61	**<0.0001**
Time × Intervention	0.035	8	0.0044	F (8, 176) = 0.48	0.87
Subject (matching)	2.65	22	0.12		
Subject × Intervention	1.06	22	0.048		
Subject × Time	2.14	176	0.012		
Residual	1.61	176	0.0092		

Incremental HDL-c (mmol/L)

	SS	*df*	MS	*F*	*p*
Intervention	0.020	1	0.020	F (1.000, 22.00) = 0.66	0.425
Time (min)	0.21	8	0.026	F (3.649, 80.27) = 8.56	**<0.0001**
Time × Intervention	0.0090	8	0.0011	F (5.813, 127.9) = 0.41	0.869
Subject (matching)	0.62	22	0.028		
Subject × Intervention	0.68	22	0.031		
Subject × Time	0.53	176	0.0030		
Residual	0.49	176	0.0028		

Incremental LDL-c (mmol/L)

	SS	*df*	MS	*F*	*p*
Intervention	0.0078	1	0.0078	F (1.000, 22.00) = 0.096	0.759
Time (min)	3.34	8	0.42	F (2.182, 48.01) = 17.81	**<0.0001**
Time × Intervention	0.058	8	0.0073	F (5.079, 111.7) = 0.074	0.597
Subject (matching)	4.58	22	0.21		
Subject × Intervention	1.79	22	0.081		
Subject × Time	4.12	176	0.023		
Residual	1.73	176	0.0098		

Incremental Apo-A1 (mmol/L)

	SS	*df*	MS	*F*	*p*
Intervention	0.052	1	0.052	F (1.000, 23.00) = 3.30	0.0822
Time (min)	0.13	4	0.032	F (3.287, 75.60) = 6.31	**0.0005**
Time × Intervention	0.016	4	0.0038	F (3.349, 77.03) = 0.77	0.529
Subject (matching)	0.43	23	0.019		
Subject × Intervention	0.36	23	0.016		
Subject × Time	0.46	92	0.0050		
Residual	0.45	92	0.0049		

Incremental Apo-B (mmol/L)					
	SS	***df***	**MS**	***F***	***p***
Intervention	0.0046	1	0.0046	F (1.000, 23.00) = 1.71	0.204
Time (min)	0.0055	4	0.0014	F (3.098, 71.26) = 1.64	0.187
Time × Intervention	0.0013	4	0.00032	F (3.008, 69.19) = 0.42	0.739
Subject (matching)	0.073	23	0.0032		
Subject × Intervention	0.063	23	0.0027		
Subject × Time	0.078	92	0.00084		
Residual	0.069	92	0.00075		

SS: sum of squares; *df*: degree of freedom; MS: mean square. Statistical significance was determined at $p < 0.05$ and is represented in bold.

Online Supporting Material 9 – Changes in postprandial lipoprotein subclasses

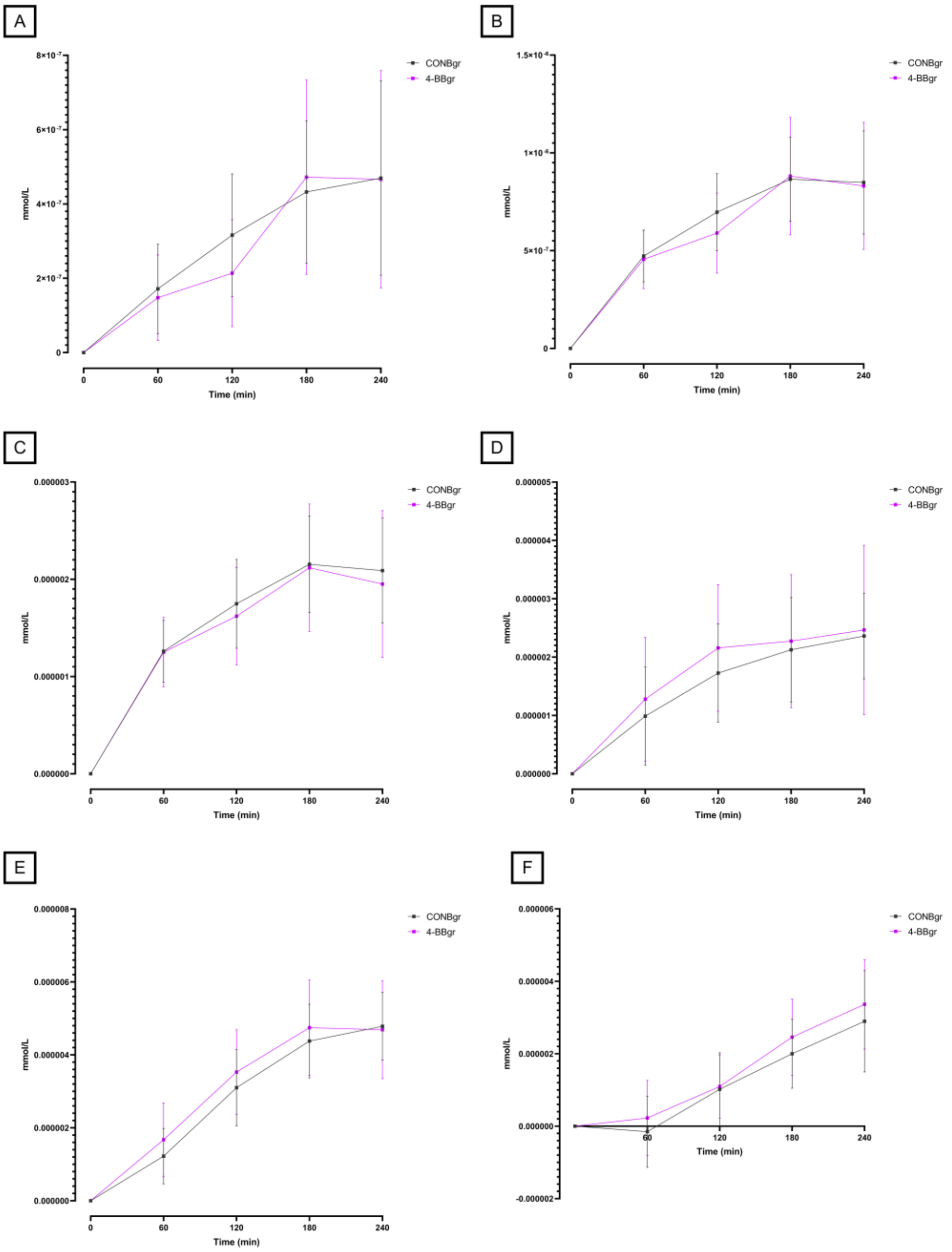

SUPPLEMENTAL FIGURE 9

Postprandial changes in lipoprotein particle responses over 4 h. **A:** CM and XXL-VLDL **B:** XL-VLDL; **C:** L-VLDL; **D:** M-VLDL; **E:** S-VLDL; **F:** XS-VLDL; **G:** IDL; **H:** L-LDL; **I:** M-LDL; **J:** S-LDL; **K:** XL-HDL; **L:** L-HDL; **M:** M-HDL; **N:** S-HDL. Values are represented as means and 95% confidence intervals.

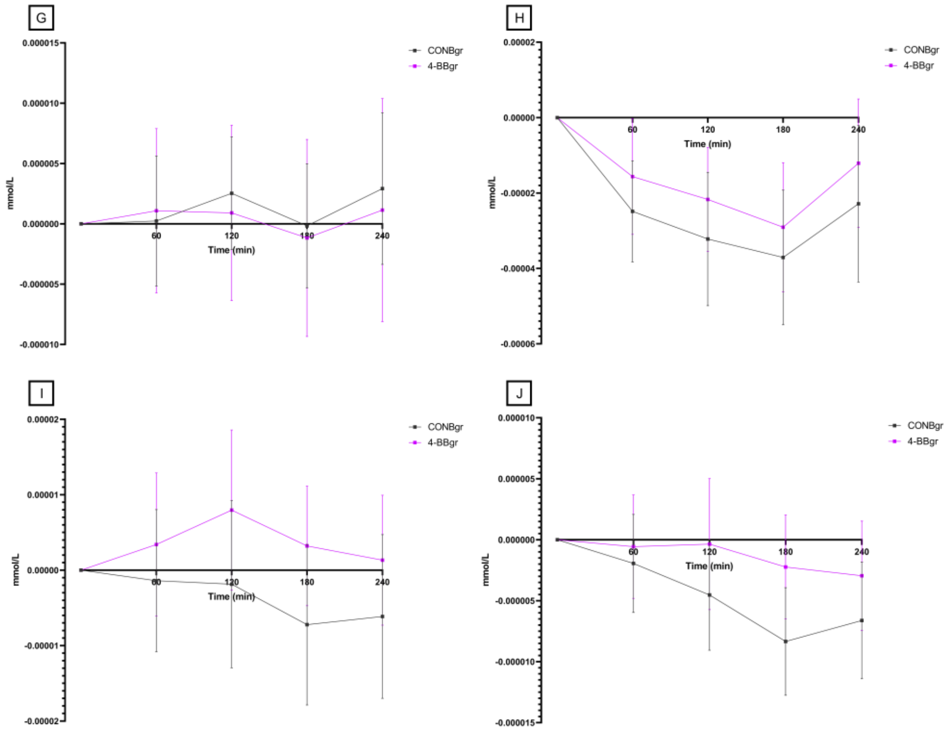

SUPPLEMENTAL FIGURE 9

(Continued)

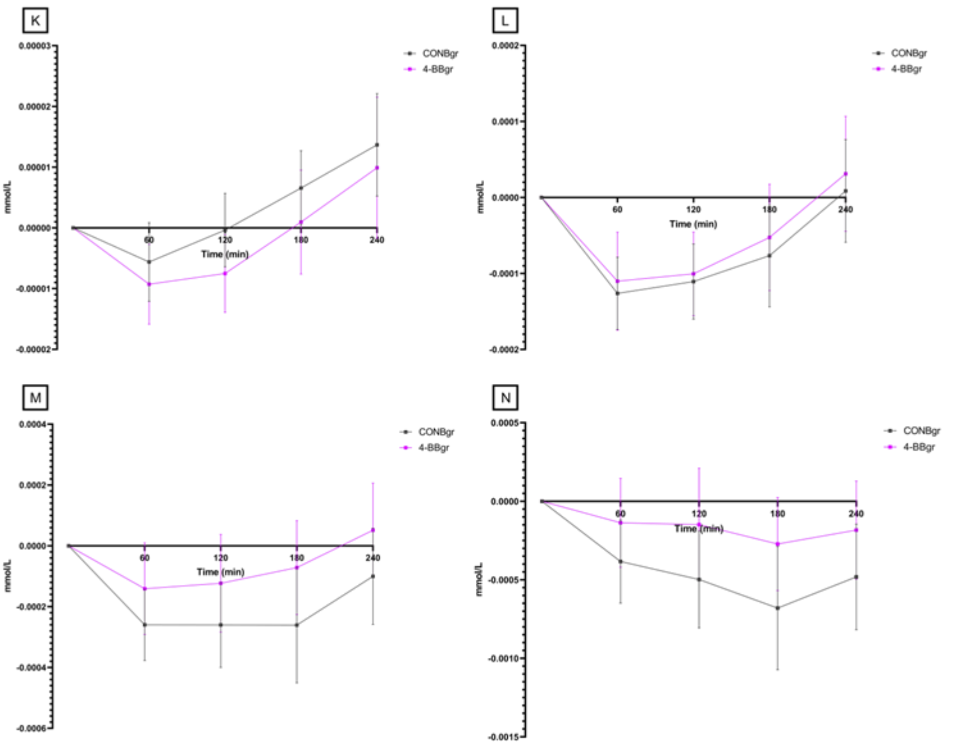

SUPPLEMENTAL FIGURE 9

(Continued)

SUPPLEMENTAL TABLE 9A

Changes in postprandial lipoprotein particle sizes over 4 h

	Lipoprotein particle sizes		
	**Diameter (nm)**		
	**CONBgr**	**4-BBgr**	***p***
VLDL	0.424 (0.25)	0.389 (0.22)	0.092
LDL	-0.0105 (0.012)	-0.0206 (0.018)	0.14
HDL	0.00629 (0.016)	0.00116 (0.018)	0.0946

p: intervention *p*-value, one-way ANOVA; p_{time}: time effect, two-way ANOVA; $p_{intervention}$: intervention effect, two-way ANOVA; $p_{interaction}$: time × intervention *p*-value, two-way ANOVA. VLDL, very low-density lipoprotein; LDL, low-density lipoprotein; HDL, high-density lipoprotein. Values are represented as means and standard deviations. Statistical significance was determined at $p < 0.05$.

SUPPLEMENTAL TABLE 9B

Changes in postprandial lipid composition of lipoprotein subclasses over 4 h

	Lipoprotein subclasses								
	**Mean concentration (mmol/L)**						**Net AUC (mmol/L*min)**		
	**CONBgr**	**4-BBgr**	***p***	p_{time}	$p_{intervention}$	$p_{interaction}$	**CONBgr**	**4-BBgr**	***p***
	CM and XXL-VLDL								
Total lipids	0.0372	0.0350	0.538	**0.0004**	0.662	0.337	9.29	8.61	0.581
TG	0.0261	0.0240	0.439	**0.0007**	0.541	0.308	6.57	5.91	0.428
PL	0.00482	0.00476	0.909	**0.0005**	0.919	0.282	1.19	1.17	0.898

TC	0.00629	0.00625	0.951	< **0.0001**	0.975	0.383	1.53	1.53	0.992
FC	0.00275	0.00265	0.765	**0.0004**	0.792	0.259	0.675	0.647	0.770
CE	0.00354	0.00361	0.835	< **0.0001**	0.931	0.739	0.850	0.882	0.871
	XL-VLDL								
Total lipid	0.0349	0.0329	0.168	< **0.0001**	0.594	0.671	8.94	8.39	0.559
TG	0.0244	0.0228	0.158	< **0.0001**	0.525	0.596	6.27	5.85	0.482
PL	0.00593	0.00558	0.276	< **0.0001**	0.587	0.468	1.52	1.41	0.535
TC	0.00460	0.00450	0.122	< **0.0001**	0.897	0.995	1.15	1.09	0.751
FC	0.00299	0.00275	0.190	< **0.0001**	0.499	0.474	0.762	0.694	0.460
CE	0.00161	0.00175	0.387	< **0.0001**	0.757	0.740	0.392	0.438	0.694
	L-VLDL								
Total lipid	0.0495	0.0462	0.0560	< **0.0001**	0.546	0.838	12.77	11.97	0.561
TG	0.0296	0.0273	**0.0409**	< **0.0001**	0.473	0.818	0.128	0.118	0.479
PL	0.00999	0.00949	0.736	< **0.0001**	0.642	0.891	2.56	2.44	0.667
TC	0.00991	0.00941	0.145	< **0.0001**	0.698	0.879	0.0423	0.0403	0.726
FC	0.00560	0.00529	0.138	< **0.0001**	0.634	0.802	0.0240	0.0227	0.649
CE	0.00432	0.00412	0.210	< **0.0001**	0.772	0.919	0.0183	0.0176	0.810
	M-VLDL								
Total lipid	0.0357	0.0387	0.0906	< **0.0001**	0.685	0.944	0.151	0.166	0.641
TG	0.0357	0.0349	0.492	< **0.0001**	0.853	0.884	0.153	0.152	0.932
PL	0.00447	0.00553	**0.0293**	< **0.0001**	0.510	0.922	0.0184	0.0233	0.487
TC	-0.00450	-0.00175	**0.0233**	0.124	0.250	0.721	-0.0206	-0.00885	0.266
FC	0.00113	0.00175	**0.0217**	**0.044**	0.531	0.946	0.00441	0.00714	0.525
CE	-0.000563	-0.00349	**0.0274**	**0.0011**	0.145	0.515	-0.0250	-0.0160	0.165
	S-VLDL								
Total lipid	0.0256	0.0295	0.0500	< **0.0001**	0.313	0.724	0.105	0.124	0.262

TG	0.0226	0.0233	0.373	**< 0.0001**	0.697	0.721	0.0949	0.0994	0.575
PL	0.00247	0.00369	**0.0310**	**< 0.0001**	0.226	0.663	0.00968	0.0154	0.199
TC	0.000542	0.00248	**0.0290**	**0.0046**	0.160	0.594	0.000709	0.00939	0.152
FC	-0.000225	0.000516	**0.0251**	0.261	0.259	0.737	-0.00145	0.00191	0.244
CE	0.000767	0.00196	**0.0328**	**0.0001**	0.108	0.499	0.00216	0.00749	0.104
	XS-VLDL								
Total lipid	0.00865	0.00936	0.365	**< 0.0001**	0.805	0.917	0.0317	0.0341	0.848
TG	0.00574	0.00644	0.0658	**< 0.0001**	0.287	0.660	0.0230	0.0263	0.236
PL	0.00219	0.00191	0.305	**< 0.0001**	0.768	0.902	0.00739	0.00588	0.712
TC	0.000728	0.00100	0.595	**0.0002**	0.852	0.810	0.00136	0.00189	0.933
FC	0.000901	0.000812	0.488	**< 0.0001**	0.850	0.904	0.00316	0.00262	0.792
CE	-0.000173	0.000192	0.382	**0.0033**	0.725	0.750	-0.00180	-0.000728	0.812

p: intervention *p*-value, one-way ANOVA; p_{time}: time effect, two-way ANOVA; $p_{intervention}$: intervention effect, two-way ANOVA; $p_{interaction}$: time × intervention *p*-value, two-way ANOVA. TG, triglycerides; PL, phospholipids; TC, total cholesterol; FC, free cholesterol; CE, cholesteryl esters. Statistical significance was determined at p <0.05 and is represented in bold.

SUPPLEMENTAL TABLE 9B (continued)

	**Lipoprotein subclasses**								
	**Mean concentration (mmol/L)**						**Net AUC (mmol/L*min)**		
	**CONBgr**	**4-BBgr**	***p***	p_{time}	$p_{intervention}$	$p_{interaction}$	**CONBgr**	**4-BBgr**	***p***
	IDL								
Total lipid	-0.0218	-0.00842	**0.0238**	0.0747	0.270	0.828	-0.177	-0.130	0.274
TG	0.00433	0.00577	**0.0428**	**< 0.0001**	0.115	0.491	0.0165	0.0230	0.0862
PL	-0.00981	-0.00627	**0.0299**	**< 0.0001**	0.202	0.639	-0.0446	-0.0305	0.230

TC	-0.0163	-0.00782	**0.0264**	0.128	0.352	0.886	-0.0740	-0.0368	0.353
FC	-0.00581	-0.00382	**0.0327**	**0.0027**	0.332	0.854	-0.0263	-0.0182	0.361
CE	-0.0105	-0.00400	**0.0384**	0.318	0.365	0.852	-0.0478	-0.0186	0.358
	L-LDL								
Total lipid	-0.0695	-0.0352	**0.0195**	**0.0001**	0.0949	0.503	-18.2	-9.31	0.0986
TG	0.00229	0.00426	**0.0444**	< **0.0001**	0.0630	0.331	0.490	1.02	**0.0466**
PL	-0.0152	-0.00812	**0.0207**	< **0.0001**	0.101	0.543	-3.94	-2.10	0.104
TC	-0.0566	-0.0314	**0.0187**	< **0.0001**	0.106	0.517	-14.8	-8.23	0.112
FC	-0.0158	-0.0102	**0.0197**	< **0.0001**	0.162	0.650	-4.08	-2.62	0.173
CE	-0.0408	-0.0212	**0.0184**	< **0.0001**	0.0921	0.474	-10.7	-5.61	0.0964
	M-LDL								
Total lipid	-0.0196	-0.00270	**0.0249**	**0.0315**	0.0510	0.412	-5.04	-0.547	**0.0458**
TG	0.00143	0.00212	0.0523	< **0.0001**	0.0886	0.347	0.338	0.525	0.0680
PL	-0.00523	-0.00137	**0.0226**	**0.0229**	0.0825	0.502	-1.34	-0.306	0.0761
TC	-0.0158	-0.00346	**0.0253**	**0.0061**	**0.0463**	0.967	-4.03	-0.766	**0.0417**
FC	-0.00752	-0.00441	**0.0197**	< **0.0001**	**0.0831**	0.472	-1.92	-1.09	0.0796
CE	-0.00826	0.000951	**0.0280**	0.1537	**0.0445**	0.396	-2.12	0.323	**0.0390**
	S-LDL								
Total lipid	-0.00744	-0.00256	**0.0293**	**0.0105**	0.102	0.489	-1.92	-0.606	0.0900
TG	0.00143	0.00164	0.108	< **0.0001**	0..286	0.471	0.355	0.412	0.255
PL	-0.00211	-0.00127	**0.0395**	**0.0008**	0.199	0.584	-0.539	-0.306	0.172
TC	-0.00675	-0.00293	**0.0289**	**0.0005**	0.0874	0.459	-1.74	-0.712	0.0782
FC	-0.00223	-0.00134	**0.0293**	< **0.0001**	0.114	0.553	-0.555	-0.316	0.106
CE	-0.00452	-0.00160	**0.0300**	**0.0039**	0.00884	0.438	-1.18	-0.396	0.0786

p: intervention *p*-value, one-way ANOVA; p_{time}: time effect, two-way ANOVA; $p_{intervention}$: intervention effect, two-way ANOVA; $p_{interaction}$: time × intervention *p*-value, two-way ANOVA. TG, triglycerides; PL, phospholipids; TC, total cholesterol; FC, free cholesterol; CE, cholesteryl esters. Statistical significance was determined at p <0.05 and is represented in bold.

SUPPLEMENTAL TABLE 9B (continued)

	**Lipoprotein subclasses**								
	**Mean concentration (mmol/L)**						**Net AUC (mmol/L*min)**		
	**CONBgr**	**4-BBgr**	***p***	p_{time}	$p_{intervention}$	$p_{interaction}$	**CONBgr**	**4-BBgr**	***p***
	XL-HDL								
Total lipid	0.00135	-0.00318	**0.0270**	**< 0.0001**	**0.0406**	0.354	0.129	-1.10	**0.0233**
TG	0.00109	0.00111	0.633	**< 0.0001**	0.868	0.821	0.267	0.273	0.867
PL	0.00150	-0.00143	**0.0273**	**< 0.0001**	**0.0281**	0.298	0.257	-0.534	**0.0159**
TC	-0.00124	-0.00287	**0.0262**	**< 0.0001**	0.0587	0.432	-0.396	-0.839	**0.0341**
FC	0.000212	-0.000528	**0.0307**	0.202	**0.0041**	0.154	0.0481	-0.155	**0.00190**
CE	-0.00145	-0.00234	**0.0240**	**< 0.0001**	0.194	0.707	-0.444	-0.684	0.152
	L-HDL								
Total lipid	-0.0255	-0.0195	**0.0380**	**< 0.0001**	0.579	0.955	-7.76	-6.24	0.586
TG	0.00457	0.00468	0.637	**< 0.0001**	0.848	0.848	1.11	1.14	0.871
PL	-0.0110	-0.00738	**0.0449**	**< 0.0001**	0.498	0.905	-3.41	-2.50	0.506
TC	-0.0191	-0.0168	**0.0216**	**< 0.0001**	0.160	0.987	-5.47	-4.88	0.667
FC	-0.00233	-0.00222	0.602	**< 0.0001**	0.924	0.964	--0.727	-0.710	0.955
CE	-0.0168	-0.0146	**0.0187**	**< 0.0001**	0.231	0.979	-4.74	-4.17	0.597
	M-HDL								
Total lipid	-0.0374	-0.00843	**0.0209**	**0.0011**	0.0586	0.455	-10.7	-3.15	0.0602

TG	0.00630	0.00698	0.131	< **0.0001**	0.365	0.l534	1.55	1.72	0.388
PL	-0.0134	-0.000645	**0.0218**	**0.001**	0.0515	0.426	-3.95	-0.631	0.0532
TC	-0.0303	-0.0148	**0.0199**	< **0.0001**	0.0693	0.485	-8.33	-4.24	0.0702
FC	-0.00412	-0.00134	**0.0208**	0.0011	0.0863	0.543	-1.17	-0.443	0.0868
CE	-0.0262	-0.0134	**0.0198**	< **0.0001**	0.0665	0.471	-7.16	-3.80	0.0675
	S-HDL								
Total lipid	-0.0303	-0.00208	**0.0201**	0.108	**0.0324**	0.360	-8.39	-0.924	**0.0321**
TG	0.0500	0.00568	0.0667	< **0.0001**	0.177	0.471	1.25	1.43	0.165
PL	-0.0145	0.000747	**0.0195**	**0.0463**	**0.0358**	0.387	-4.12	-0.125	**0.0361**
TC	-0.0209	-0.00851	**0.0208**	**0.0012**	**0.0352**	0.347	-5.51	-2.23	**0.0342**
FC	-0.00263	0.000218	**0.0209**	0.204	**0.0431**	0.424	-0.743	0.0158	**0.0405**
CE	-0.0183	-0.00873	**0.0209**	< **0.0001**	**0.0345**	0.329	-4.77	-2.24	**0.0338**

p: intervention p-value, one-way ANOVA; p_{time}: time effect, two-way ANOVA; $p_{intervention}$: intervention effect, two-way ANOVA; $p_{interaction}$: time × intervention p-value, two-way ANOVA. TG, triglycerides; PL, phospholipids; TC, total cholesterol; FC, free cholesterol; CE, cholesteryl esters. Statistical significance was determined at p <0.05 and is represented in bold.

SUPPLEMENTAL TABLE 9C

Two-way ANOVA summary table for the changes in postprandial lipoprotein and lipoprotein subfractional particle concentrations over 4 h

	SS	*df*	MS	*F*	*p*
**Incremental VLDL (mmol/L)**					
Intervention	2.13×10^{-11}	1	2.13×10^{-11}	F (1, 23) = 0.23	0.639
Time (min)	6.16×10^{-9}	4	1.54×10^{-9}	F (3.17, 72.79) = 48.58	**<0.0001**
Time × Intervention	9.82×10^{-12}	4	2.45×10^{-12}	F (3.24, 74.53) = 0.10	0.967
Subject (matching)	3.96×10^{-9}	23	1.72×10^{-10}		
Subject × Intervention	2.17×10^{-9}	23	9.44×10^{-11}		
Subject × Time	2.92×10^{-9}	92	3.17×10^{-11}		
Residual	2.26×10^{-9}	92	2.46×10^{-11}		
**Incremental CM and XXL-VLDL (mmol/L)**					
	SS	*df*	MS	*F*	*p*
Intervention	1.92×10^{-14}	1	1.92×10^{-14}	F (1, 23) = 0.29	0.595
Time (min)	7.56×10^{-12}	4	1.89×10^{-12}	F (1.64, 37.77) = 11.75	**0.0003**
Time × Intervention	1.33×10^{-13}	4	3.32×10^{-14}	F (2.17, 49.89) = 1.17	0.323
Subject (matching)	2.42×10^{-11}	23	1.05×10^{-12}		
Subject × Intervention	1.52×10^{-12}	23	6.61×10^{-14}		
Subject × Time	1.48×10^{-11}	92	1.61×10^{-13}		
Residual	2.62×10^{-12}	92	2.84×10^{-14}		
**Incremental XL-VLDL (mmol/L)**					
	SS	*df*	MS	*F*	*p*
Intervention	3.82×10^{-14}	1	3.82×10^{-14}	F (1.000, 23.00) = 0.1832	0.6726
Time (min)	2.43×10^{-11}	4	6.08×10^{-12}	F (1.689, 38.84) = 31.83	**<0.0001**
Time × Intervention	1.10×10^{-13}	4	2.75×10^{-14}	F (2.563, 58.95) = 0.5598	0.6167
Subject (matching)	2.89×10^{-11}	23	1.25×10^{-12}		
Subject × Intervention	4.79×10^{-12}	23	2.09×10^{-13}		
Subject × Time	1.76×10^{-11}	92	1.91×10^{-13}		
Residual	4.52×10^{-12}	92	4.91×10^{-14}		
**Incremental L-VLDL (mmol/L)**					
	SS	*df*	MS	*F*	*p*
Intervention	2.33×10^{-13}	1	2.33×10^{-13}	F (1.000, 23.00) = 0.17	0.684
Time (min)	1.43×10^{-10}	4	3.58×10^{-11}	F (1.873, 43.08) = 36.07	**<0.0001**

Time × Intervention	2.11×10^{-13}	4	5.27×10^{-14}	F (2.652, 61.00) = 0.18	0.892
Subject (matching)	1.36×10^{-10}	23	5.93×10^{-12}		
Subject × Intervention	3.16×10^{-11}	23	1.37×10^{-12}		
Subject × Time	9.14×10^{-11}	92	9.94×10^{-13}		
Residual	2.75×10^{-11}	92	2.99×10^{-13}		
**Incremental M-VLDL (mmol/L)**					
	SS	***df***	**MS**	***F***	***p***
Intervention	2.27×10^{-12}	1	2.27×10^{-12}	F (1, 23) = 0.23	0.636
Time (min)	1.87×10^{-10}	4	4.68×10^{-11}	F (4, 92) = 13.98	**<0.0001**
Time × Intervention	1.37×10^{-12}	4	3.41×10^{-13}	F (4, 92) = 0.14	0.966
Subject (matching)	3.37×10^{-10}	23	1.47×10^{-11}		
Subject × Intervention	2.28×10^{-10}	23	9.89×10^{-12}		
Subject × Time	3.08×10^{-10}	92	3.35×10^{-12}		
Residual	2.20×10^{-10}	92	2.39×10^{-12}		
**Incremental S-VLDL (mmol/L)**					
	SS	***df***	**MS**	***F***	***p***
Intervention	3.18×10^{-10}	1	3.18×10^{-12}	F (1, 23) = 0.42	0.523
Time (min)	8.06×10^{-10}	4	2.02×10^{-10}	F (2.71, 62.24) = 51.19	**<0.0001**
Time × Intervention	3.16×10^{-12}	4	7.90×10^{-13}	F (3.20, 73.63) = 0.37	0.790
Subject (matching)	4.80×10^{-10}	23	2.09×10^{-11}		
Subject × Intervention	1.74×10^{-10}	23	7.55×10^{-12}		
Subject × Time	3.62×10^{-10}	92	3.94×10^{-12}		
Residual	1.98×10^{-10}	92	2.15×10^{-12}		
**Incremental XS-VLDL (mmol/L)**					
	SS	***df***	**MS**	***F***	***p***
Intervention	4.58×10^{-12}	1	4.58×10^{-12}	F (1, 23) = 0.44	0.512
Time (min)	3.63×10^{-10}	4	9.07×10^{-11}	F (2.72, 62.64) = 29.17	**<0.0001**
Time × Intervention	2.36×10^{-12}	4	5.89×10^{-13}	F (3.54, 81.46) = 0.21	0.918
Subject (matching)	4.16×10^{-10}	23	1.81×10^{-11}		
Subject × Intervention	2.37×10^{-10}	23	1.03×10^{-11}		
Subject × Time	2.86×10^{-10}	92	3.11×10^{-12}		
Residual	2.64×10^{-10}	92	2.87×10^{-12}		

SS: sum of squares; *df*: degree of freedom; MS: mean square. Statistical significance was determined at $p < 0.05$ and is represented in bold.

SUPPLEMENTAL TABLE 9C (Continued)

Incremental IDL (mmol/L)					
	SS	*df*	MS	*F*	*p*
Intervention	0.0016	1	0.0016	F (1, 23) = 0.99	0.331
Time (min)	0.053	4	0.013	F (2.6, 60.11) = 15.90	**<0.0001**
Time × Intervention	0.0013	4	0.0003	F (2.8, 64.42) = 0.77	0.505
Subject (matching)	0.11	23	0.0046		
Subject × Intervention	0.037	23	0.0016		
Subject × Time	0.076	92	0.00083		
Residual	0.040	92	0.00043		

Incremental LDL (mmol/L)					
	SS	*df*	MS	*F*	*p*
Intervention	1.79×10^{-8}	1	1.79×10^{-8}	F (1, 23) = 2.93	0.100
Time (min)	4.08×10^{-8}	4	1.02×10^{-8}	F (2.92, 67.12) = 5.03	**0.0036**
Time × Intervention	5.14×10^{-9}	4	1.28×10^{-9}	F (3, 68.98) = 0.79	0.506
Subject (matching)	1.86×10^{-7}	23	8.08×10^{-9}		
Subject × Intervention	1.40×10^{-7}	23	6.10×10^{-9}		
Subject × Time	1.87×10^{-7}	92	2.03×10^{-9}		
Residual	1.50×10^{-7}	92	1.64×10^{-9}		

Incremental L-LDL (mmol/L)					
	SS	*df*	MS	*F*	*p*
Intervention	3.56×10^{-9}	1	3.56×10^{-9}	F (1, 23) = 1.77	0.196
Time (min)	3×10^{-8}	4	7.50×10^{-9}	F (2.71, 62.40) = 7.68	**0.0003**
Time × Intervention	9.46×10^{-10}	4	2.37×10^{-10}	F (3.36, 77.19) = 0.475	0.722
Subject (matching)	1.09×10^{-7}	23	4.73×10^{-9}		
Subject × Intervention	4.62×10^{-8}	23	2.01×10^{-9}		
Subject × Time	8.99×10^{-8}	92	9.77×10^{-10}		
Residual	4.59×10^{-8}	92	4.99×10^{-10}		

Incremental M-LDL (mmol/L)					
	SS	*df*	MS	*F*	*p*
Intervention	2.53×10^{-9}	1	2.53×10^{-9}	F (1, 23) = 3.50	0.074
Time (min)	9.65×10^{-10}	4	2.41×10^{-10}	F (3.46, 79.51) = 0.902	0.456
Time × Intervention	8.70×10^{-10}	4	2.18×10^{-10}	F (2.86, 65.86) = 0.758	0.516
Subject (matching)	3.33×10^{-8}	23	1.45×10^{-9}		
Subject × Intervention	1.66×10^{-8}	23	7.23×10^{-10}		

	SS	*df*	MS		
Subject × Time	2.46×10^{-8}	92	2.68×10^{-10}		
Residual	2.64×10^{-8}	92	2.87×10^{-10}		

**Incremental S-LDL (mmol/L)**					
	SS	***df***	**MS**	***F***	***p***
Intervention	5.63×10^{-10}	1	5.63×10^{-10}	F (1, 23) = 2.80	0.108
Time (min)	9.84×10^{-10}	4	2.46×10^{-10}	F (3.65, 83.89) = 4.26	**0.0045**
Time × Intervention	2.77×10^{-10}	4	6.91×10^{-11}	F (2.75, 63.14) = 1.13	0.343
Subject (matching)	5.58×10^{-9}	23	2.43×10^{-10}		
Subject × Intervention	4.62×10^{-9}	23	2.01×10^{-10}		
Subject × Time	5.32×10^{-9}	92	5.78×10^{-11}		
Residual	5.656e-009	92	6.148e-011		

SS: sum of squares; *df*: degree of freedom; MS: mean square. Statistical significance was determined at p <0.05 and is represented in bold.

SUPPLEMENTAL TABLE 9C (Continued)

**Incremental HDL (mmol/L)**					
	SS	***df***	**MS**	***F***	***p***
Intervention	9.13×10^{-6}	1	9.13×10^{-6}	F (1, 23) = 3.86	0.0617
Time (min)	1.59×10^{-5}	4	3.97×10^{-6}	F (3.41, 78.46) = 5.11	**0.0018**
Time × Intervention	2.63×10^{-6}	4	6.57×10^{-7}	F (3.37, 77.43) = 0.903	0.453
Subject (matching)	7.24×10^{-5}	23	3.15×10^{-6}		
Subject × Intervention	5.44×10^{-5}	23	2.37×10^{-6}		
Subject × Time	7.15×10^{-5}	92	7.77×10^{-7}		
Residual	6.70×10^{-5}	92	7.28×10^{-7}		
**Incremental XL-HDL (mmol/L)**					
	SS	***df***	**MS**	***F***	***p***
Intervention	9.76×10^{-10}	1	9.76×10^{-10}	F (1, 23) = 2.2	0.148
Time (min)	1.06×10^{-8}	4	2.65×10^{-9}	F (3.05, 70.16) = 13.70	**<0.0001**
Time × Intervention	3.43×10^{-10}	4	8.58×10^{-11}	F (2.99, 68.64) = 0.64	0.589
Subject (matching)	2.20×10^{-8}	23	9.55×10^{-10}		
Subject × Intervention	1.00×10^{-8}	23	4.36×10^{-10}		
Subject × Time	1.78×10^{-8}	92	1.93×10^{-10}		
Residual	1.23×10^{-8}	92	1.33×10^{-10}		
**Incremental L-HDL (mmol/L)**					
	SS	***df***	**MS**	***F***	***p***

Intervention	1.27×10^{-8}	1	1.27×10^{-8}	F (1, 23) = 0.30	0.586
Time (min)	7.32×10^{-7}	4	1.83×10^{-7}	F (3.13, 71.98) = 15.21	**<0.0001**
Time × Intervention	4.63×10^{-9}	4	1.16×10^{-9}	F (3.38, 77.65) = 0.11	0.967
Subject (matching)	1.01×10^{-6}	23	4.40×10^{-8}		
Subject × Intervention	9.59×10^{-7}	23	4.17×10^{-8}		
Subject × Time	1.11×10^{-6}	92	1.20×10^{-8}		
Residual	9.99×10^{-7}	92	1.09×10^{-8}		
**Incremental M-HDL (mmol/L)**					
	SS	***df***	**MS**	***F***	***p***
Intervention	8.54×10^{-7}	1	8.54×10^{-7}	F (1, 23) = 3.86	0.0616
Time (min)	1.79×10^{-6}	4	4.46×10^{-7}	F (3.26, 74.91) = 6.17	**0.0006**
Time × Intervention	2.45×10^{-7}	4	6.14×10^{-8}	F (3.36, 77.24) = 0.88	0.468
Subject (matching)	6.40×10^{-6}	23	2.78×10^{-7}		
Subject × Intervention	5.09×10^{-6}	23	2.21×10^{-7}		
Subject × Time	6.66×10^{-6}	92	7.24×10^{-8}		
Residual	6.45×10^{-6}	92	7.01×10^{-8}		
**Incremental S-HDL (mmol/L)**					
	SS	***df***	**MS**	***F***	***p***
Intervention	4.07×10^{-6}	1	4.07×10^{-6}	F (1, 23) = 4.94	**0.0364**
Time (min)	5.85×10^{-6}	4	1.46×10^{-6}	F (3.37, 77.52) = 4.61	**0.0037**
Time × Intervention	1.19×10^{-6}	4	2.97×10^{-7}	F (3.37, 77.57) = 1.13	0.345
Subject (matching)	3.42×10^{-5}	23	1.49×10^{-6}		
Subject × Intervention	1.90×10^{-5}	23	8.24×10^{-7}		
Subject × Time	2.92×10^{-5}	92	3.18×10^{-7}		
Residual	2.42×10^{-5}	92	2.63×10^{-7}		

SS: sum of squares; *df*: degree of freedom; MS: mean square. Statistical significance was determined at $p < 0.05$ and is represented in bold.
