# Supplementary material for: Postprandial glycemic and lipidemic effects of black rice anthocyanin extract fortification in foods of varying macronutrient compositions and matrices
Source: NPJ Sci Food. 2023 Nov 1;7:59. doi: 10.1038/s41538-023-00233-y (PMC10620212; doi:10.1038/s41538-023-00233-y)
Supplement: Supplementary file 2 — Reporting summary [file 41538_2023_233_MOESM2_ESM.pdf]

Corresponding author(s): Dr. Mei Hui LIU

Last updated by author(s): Jul 12, 2023

Reporting Summary

Nature Portfolio wishes to improve the reproducibility of the work that we publish. This form provides structure for consistency and transparency in reporting. For further information on Nature Portfolio policies, see our Editorial Policies and the Editorial Policy Checklist.

Statistics

For all statistical analyses, confirm that the following items are present in the figure legend, table legend, main text, or Methods section.

n/a	Confirmed	
☐	☒	The exact sample size (*n*) for each experimental group/condition, given as a discrete number and unit of measurement
☐	☒	A statement on whether measurements were taken from distinct samples or whether the same sample was measured repeatedly
☐	☒	The statistical test(s) used AND whether they are one- or two-sided<br>*Only common tests should be described solely by name; describe more complex techniques in the Methods section.*
☒	☐	A description of all covariates tested
☐	☒	A description of any assumptions or corrections, such as tests of normality and adjustment for multiple comparisons
☐	☒	A full description of the statistical parameters including central tendency (e.g. means) or other basic estimates (e.g. regression coefficient) AND variation (e.g. standard deviation) or associated estimates of uncertainty (e.g. confidence intervals)
☐	☒	For null hypothesis testing, the test statistic (e.g. *F*, *t*, *r*) with confidence intervals, effect sizes, degrees of freedom and *P* value noted<br>*Give P values as exact values whenever suitable.*
☒	☐	For Bayesian analysis, information on the choice of priors and Markov chain Monte Carlo settings
☒	☐	For hierarchical and complex designs, identification of the appropriate level for tests and full reporting of outcomes
☒	☐	Estimates of effect sizes (e.g. Cohen's *d*, Pearson's *r*), indicating how they were calculated

Our web collection on statistics for biologists contains articles on many of the points above.

Software and code

Policy information about availability of computer code

Data collection	No software was used for data collection.
Data analysis	Data analysis was performed using GraphPad Prism 9.0 (GraphPad, San Diego, CA, USA).

For manuscripts utilizing custom algorithms or software that are central to the research but not yet described in published literature, software must be made available to editors and reviewers. We strongly encourage code deposition in a community repository (e.g. GitHub). See the Nature Portfolio guidelines for submitting code & software for further information.

Data

Policy information about availability of data

All manuscripts must include a data availability statement. This statement should provide the following information, where applicable:

- Accession codes, unique identifiers, or web links for publicly available datasets
- A description of any restrictions on data availability
- For clinical datasets or third party data, please ensure that the statement adheres to our policy

Data described in the manuscript will be made available upon request, pending application to the corresponding author.

Research involving human participants, their data, or biological material

Policy information about studies with human participants or human data. See also policy information about sex, gender (identity/presentation), and sexual orientation and race, ethnicity and racism.

Reporting on sex and gender	No sex and gender-based analyses were conducted as it was not a primary aim of the study. There was also an insufficient sample size to stratify the subjects based on sex and gender.
Reporting on race, ethnicity, or other socially relevant groupings	The classification term "Asian Chinese" was provided by the researchers for the recruitment process. This was defined geographically, referring to participants who were of Chinese descent and were residing in Asia.
Population characteristics	1) Bread trial<br>Age: 25 (6); gender (M/F): 7/15; body mass index: 21.6 (2.5)<br>2) Burger trial<br>Age: 29 (11); gender (M/F): 12/12; body mass index: 21.3 (1.7)
Recruitment	Participants were recruited via posters and personal communication on the National University of Singapore campus grounds. Recruited participants were largely made up of students and campus staff, and hence may not be fully representative of all Asian Chinese.
Ethics oversight	National Healthcare Group

Note that full information on the approval of the study protocol must also be provided in the manuscript.

Field-specific reporting

Please select the one below that is the best fit for your research. If you are not sure, read the appropriate sections before making your selection.

☒ Life sciences ☐ Behavioural & social sciences ☐ Ecological, evolutionary & environmental sciences

For a reference copy of the document with all sections, see nature.com/documents/nr-reporting-summary-flat.pdf

Life sciences study design

All studies must disclose on these points even when the disclosure is negative.

Sample size	24 participants for each trial
Data exclusions	2 participants excluded from the bread trial as they did not complete the study procedures. None excluded from the burger trial.
Replication	Replicates of blood biochemical measurements were not possible as this would require the collection of more blood from participants. In-vitro experiments were replicated thrice, with results corroborating with each other.
Randomization	Participants were randomised into crossover groups using an on-line randomiser.
Blinding	Investigators were not blinded as the food-based interventions were visually very distinct and identifiable from each other.

Reporting for specific materials, systems and methods

We require information from authors about some types of materials, experimental systems and methods used in many studies. Here, indicate whether each material, system or method listed is relevant to your study. If you are not sure if a list item applies to your research, read the appropriate section before selecting a response.

Materials & experimental systems

n/a	Involved in the study	
☒	☐	Antibodies
☒	☐	Eukaryotic cell lines
☒	☐	Palaeontology and archaeology
☒	☐	Animals and other organisms
☐	☒	Clinical data
☒	☐	Dual use research of concern
☒	☐	Plants

Methods

n/a	Involved in the study	
☒	☐	ChIP-seq
☒	☐	Flow cytometry
☒	☐	MRI-based neuroimaging

Clinical data

Policy information about clinical studies

All manuscripts should comply with the ICMJE guidelines for publication of clinical research and a completed CONSORT checklist must be included with all submissions.

Clinical trial registration	NCT03989674 and NCT04063137
Study protocol	Full study protocols are accessible to study investigators via the National Healthcare Group online research portal. A copy of the protocol can be provided upon request to our study team.
Data collection	Participant recruitment was carried out over the course of 2-3 months for each study. Recruitment procedures take place in a private setting in a consultation room, while providing as much time as needed for participants to decide on their study participation. Sample collection was carried out in phlebotomy wards during each study visit. Phlebotomy was carried out on the arm not used for blood pressure measurements, using a butterfly cannula. Blood samples for plasma glucose measurements were collected in sodium fluoride tubes and were inverted 6 – 8 times post collection. Blood samples for serum insulin and lipid panel measurements were collected in plain tubes and left to clot for 30 minutes. Blood samples for bioavailability analysis were collected in EDTA tubes and inverted 6 – 8 times after collection. Plasma was then separated from these samples by centrifugation at 3500 × g for 15 min. Deidentified samples were sent immediately to a local diagnostic lab (Quest Laboratories) for analysis.
Outcomes	Blood glucose measurements from the Bread trial were used for the determination of GI in accordance with ISO 26642:2010. Time-series blood glucose curves of the 50-g glucose standard, CON, 2-BB, and 4-BB were constructed to obtain glucose IAUCs via the trapezoid rule. The GI of CON, 2-BB and 4-BB were determined by dividing their respective IAUCs by that of the glucose standard. The GI of each bread was expressed as the mean and standard deviation from the respective average GIs of 24 participants. Blood samples for glucose, insulin, and the lipid panel were sent to Quest Laboratories, Singapore, for biochemical analysis. Plasma glucose was determined spectrophotometrically by the hexokinase/glucose-6-phosphate dehydrogenase method. Serum insulin was determined by the electrochemiluminescence immunoassay method. Serum triglycerides (TG) were determined by colorimetry using the Fossati 3-step enzymatic reaction involving lipase, glycerol kinase, and glycerol-3-phosphate-oxidase, with a Trinder endpoint reaction. Total cholesterol (TC) was determined by colorimetry using the cholesterol esterase/cholesterol oxidase method with a Trinder endpoint reaction. High-density lipoprotein (HDL) cholesterol was determined by the Trinder colorimetric method. LDL cholesterol was calculated using the Friedewald equation. For these measurements, mean concentrations and IAUCs were determined.<br><br>Bioavailability analysis was performed in the Bread trial according to the methods by Kay, et al.. Plasma ACNs and their metabolites were extracted using C18 solid-phase extraction (SPE) cartridges (400020, Cayman Chemical, Michigan, USA) that had been preconditioned with 7 mL acidified methanol (0.1% v/v trifluoroacetic acid, pH 2.1) and then with 7 mL acidified water (10 mM oxalic acid, pH 2.2). Blood plasma was acidified with 40 uL 6 M HCl, diluted in an equal volume of 10 mM oxalic acid, vortexed and loaded directly into the SPE cartridge. The sample was drained off in a vacuum manifold and washed with 2 volumes of acidified water. The remaining ACN extract was eluted with 6 mL acidified methanol, and subsequently brought to dryness at 25 oC under a continuous stream of nitrogen gas. The residue was dissolved in 200 uL of 0.1% v/v formic acid and filtered through a 13 mm 0.22 um PTFE hydrophilic syringe filter. The resulting solution was analyzed using a reverse-phased C18 Sunfire column (250 x 4.6 mm/5 µm; Waters, Wexford, Ireland) on a HPLC system (Shimadzu Prominence, Shimadzu, Kyoto, Japan) connected with a diode array detector (DAD). The injection volume was 50 µL. The flow rate and oven temperature were maintained at 1 mL/min at 25oC. A gradient elution process was applied (mobile phase A: 5% v/v formic acid; mobile phase B: 100% acetonitrile): 0% B for 5 min, 10% B at 20 min, 13% B at 40 min, 20% B at 44 min, 25% B at 50 min, and 100% B at 55 min. Detection of ACNs was performed at 520 nm; while its metabolites, ferulic acid, vanillic acid, and protocatechuic acid were detected at 280 nm. Identification and quantification of each ACN and metabolite was based on matching the retention time and peak areas with the external calibration curve of respective standards.<br><br>Lipoprotein measurements were quantified in the Burger trial by the Nightingale high-throughput nuclear magnetic resonance (NMR) platform (Nightingale Health, Helsinki, Finland) according to previously described methods. The investigators at Nightingale Health were blinded prior to the sample analysis, which was performed in one batch to eliminate batch effects. The biomarkers measured included apolipoprotein concentrations, lipoprotein particle sizes, as well as plasma concentrations of total and subfractions of lipoproteins.
